# Tracker Nanocatalyst for Screening of Intracellular Copper‐Catalyzed Azide‐Alkyne Cycloadditions

**DOI:** 10.1002/smll.202506185

**Published:** 2025-09-16

**Authors:** Mónica Rodríguez‐Segura, Francisco Javier López‐Delgado, María Victoria Cano‐Cortés, Antonio Delgado‐González, Juan Jose Diaz‐Mochon, Rosario Maria Sanchez‐Martin

**Affiliations:** ^1^ Department of Medicinal and Organic Chemistry Excellence Research Unit of Chemistry Applied to Biomedicine and the Environment School of Pharmacy University of Granada Campus Cartuja s/n Granada 18071 Spain; ^2^ GENYO Centre for Genomics and Oncological Research Pfizer University of Granada Andalusian Regional Government PTS Granada Avenida de la Ilustración, 114 Granada 18016 Spain; ^3^ Instituto de Investigación Biosanitaria ibs.GRANADA Granada 18012 Spain; ^4^ DESTINA Genómica S.L PTS Granada Avenida de la Innovación 1, Edificio BIC Armilla 18100 Spain; ^5^ Present address: Department of Microbiology and Immunology Stanford School of Medicine, Stanford University Stanford CA 94306 USA

**Keywords:** CuAAC reactions, click chemistry, heterogeneous copper nanocatalysts, intracellular catalysis, nano‐tracker

## Abstract

Intracellular copper‐catalyzed azide‐alkyne cycloaddition (CuAAC) offers immense potential for bioorthogonal chemistry, but its application is severely hindered by copper toxicity and the challenge of controlling catalysis within the complex cellular environment. Heterogeneous copper catalysts can reduce toxicity by minimizing free copper exposure and enabling localized activity, yet optimizing their performance in situ within living cells remains a significant hurdle. Here, the development of a novel dual‐functional nanocatalyst, Cu@BTTAA‐Cy5‐NPs, that combines robust heterogeneous CuAAC catalytic activity with intrinsic fluorescence tracking is reported. The successful synthesis and characterization of these monodispersed nanoparticles is demonstrated, confirming efficient copper loading stabilized by BTTAA and the nanoparticle matrix, and critically, the retention of Cy5 fluorescence for tracking. This unique dual functionality allows for real‐time monitoring of nanoparticle localization and correlation with catalytic product formation via distinct fluorescence channels, enabling, for the first time to our knowledge, comprehensive in situ screening and optimization of CuAAC reaction conditions directly within living cells using fluorescence feedback. The nanoparticles exhibit excellent biocompatibility and cellular uptake, showing no significant toxicity, apoptosis, or oxidative stress at active concentrations.

## Graphical abstract

Development of a dual tracker nanocatalyst that represents a widely applicable advance for precise localization of catalytic reactions and real‐time screening and tuning of reaction conditions within cells.

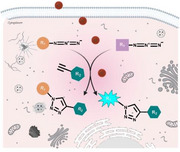

## 1 Introduction

Since the introduction of click chemistry reactions at the beginning of the twentieth century by Sharpless and Meldal and their subsequent implementation in living systems to enable bioorthogonal chemistry by Bertozzi, these reactions have profoundly impacted diverse fields, particularly chemical biology.^[^ ^1^, ^2^, ^3^, ^4^, ^5^, ^6^, ^7^, ^8^, ^9^ ^]^ The copper‐catalyzed azide‐alkyne cycloaddition (CuAAC) reaction stands as a seminal example, recognized by the Nobel Prize in Chemistry in 2022 for its efficiency and modularity in forming stable triazoles.^[^ ^10^, ^11^ ^]^ The power of CuAAC lies in its bioorthogonality – the ability to proceed efficiently and selectively within complex biological environments without interfering with native biochemical processes. This has led to its successful application in various biological contexts, including intracellular prodrug activation.^[^ ^12^, ^13^, ^14^, ^15^ ^]^

However, the use of copper in biological systems, particularly in the intracellular environment, presents significant challenges.

The bioavailability, ligand transfer, cellular uptake, and ultimately the toxicity of copper species are highly dependent on their structure and oxidation state.^[^ ^4^, ^16^ ^]^ Furthermore, the generation of reactive oxygen species (ROS) by copper catalysts can severely limit the suitability of CuAAC for sensitive biological applications.^[^ ^16^ ^]^ Effectively controlling copper localization, concentration, and activity within the dynamic and reducing environment of living cells remains a critical obstacle for widespread intracellular CuAAC applications.

To mitigate copper‐related toxicity and improve control, various strategies have been explored. These include the development of more biocompatible copper complexes and the adoption of strain‐promoted azide‐alkyne cycloaddition (SPAAC), which entirely bypasses the need for copper.^[^ ^13^, ^17^ ^]^ While SPAAC eliminates copper toxicity, it is often slower and less broadly applicable than CuAAC. Biocompatible copper complexes offer improved cellular compatibility but typically function as homogeneous catalysts, lacking the advantages of heterogeneous systems for controlled delivery, localization, and potential reusability. More recently, heterogeneous catalysts, including single‐chain metal‐organic nanoparticles, self‐adapting metal‐organic frameworks, and polymeric nanoparticles, have been developed to facilitate bioorthogonal catalysis within cells, improving copper delivery and activity control.^[^ ^18^, ^19^, ^20^, ^21^, ^22^, ^23^, ^24^, ^25^, ^26^ ^]^ For instance, copper‐loaded solid supports have been used for extracellular prodrug activation, and nanoparticle‐based systems have shown promise for intracellular reactions.^[^ ^25^, ^26^ ^]^ While these advances have significantly improved copper delivery and reduced systemic toxicity, a key limitation remains: the ability to real‐time monitor and dynamically optimize catalytic reaction conditions in situ within the complex and heterogeneous environment of living cells. Traditional approaches rely on optimizing bulk in vitro reactions, which may not accurately reflect intracellular conditions or account for cell‐to‐cell variability.

Addressing this critical gap requires a system that provides both catalytic activity and a means of tracking and correlating catalyst presence with reaction outcome at the cellular level. Inspired by the latest advancements in using CuAAC reactions in live biological systems and building on our previous development of metallofluorescent cross‐linked polystyrene nanoparticles for multimodal applications or cell barcoding, which showed the potential of embedding both metals and fluorophores within a nanoparticle platform,^[^ ^27^, ^28^, ^29^ ^]^ we aimed to create a system specifically designed for intracellular CuAAC optimization. Our previous work demonstrated that cyanine dyes covalently conjugated to polystyrene nanoparticles can chelate metals like palladium and gold, facilitating their delivery without significant cytotoxicity due to minimized metal leaching.^[^ ^27^, ^30^ ^]^ A logical extension was to explore the use of copper ions within a similar framework to enable cytoplasmic catalysis with reduced biotoxicity. The coordination of copper ions with cyanine molecules is well‐established and can influence their photophysical properties, potentially through interaction with functional groups and aromatic π‐systems of the nanoparticle matrix. In particular, coordination is expected to occur primarily via the sulfonate group, followed by secondary *π*–*π* interactions between the aromatic moieties of the cyanine dye and the aromatic domains of the nanoparticle matrix.^[^ ^31^, ^32^ ^]^

Herein, we report the development and characterization of a novel dual‐functional tracker nanocatalyst, Cu@BTTAA‐Cy5‐NPs, based on cross‐linked polystyrene nanoparticles functionalized with both the copper(I)‐stabilizing ligand BTTAA and the fluorescent dye Cy5. This unique design aims to combine robust heterogeneous copper‐catalyzed CuAAC activity with intrinsic fluorescence tracking capabilities within a single nanoparticle. We hypothesized that this integrated approach would enable, for the first time, the ability to real‐time monitor nanoparticle localization and correlate it with catalytic product formation, thereby allowing for comprehensive in situ screening and optimization of catalytic reaction conditions directly within living cells.

## 2 Results and Discussion

### 2.1 Synthesis, Functionalization, and Characterization of Fluorescent Copper Nanocatalyst

**Figure 1:**
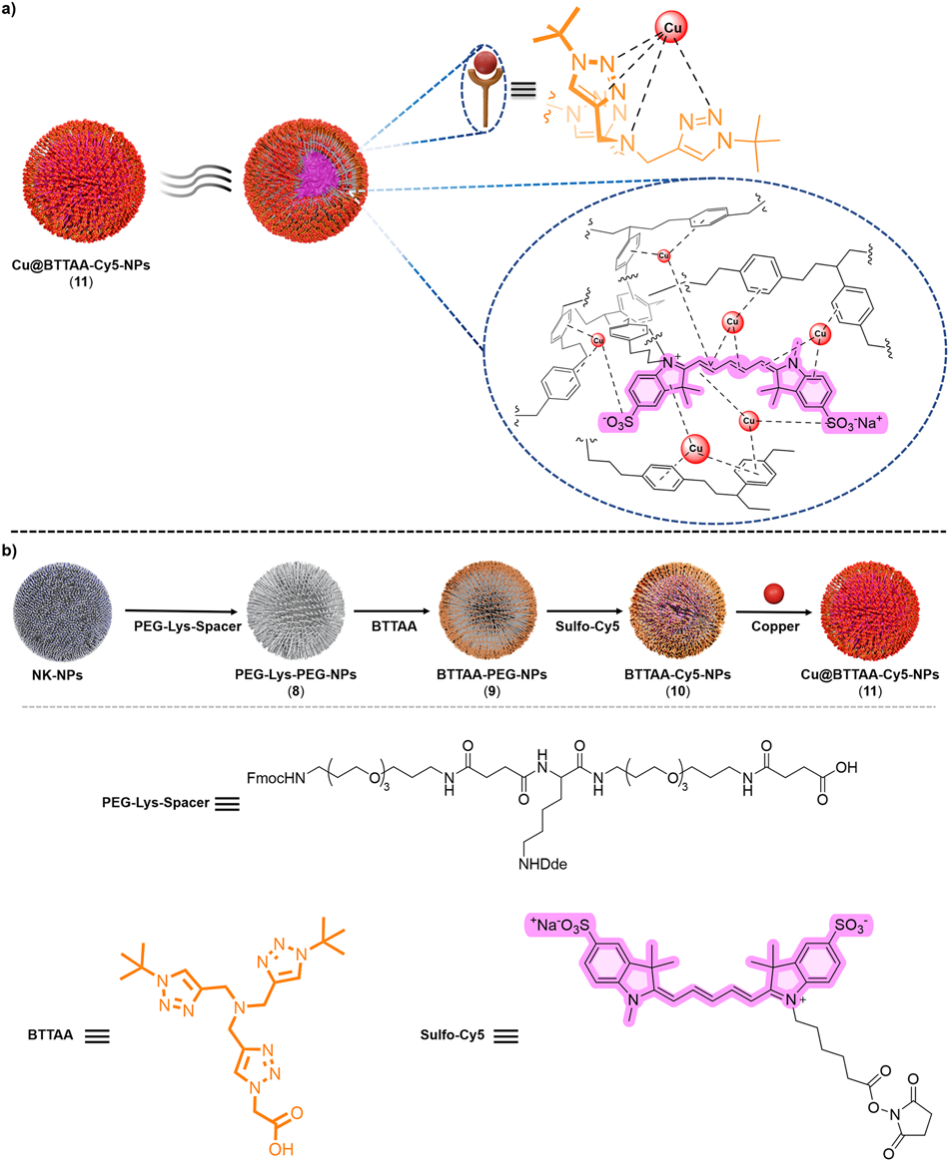
a) Schematic representation of the design of dual functionalized nanoparticles Cu@BTTAA‐Cy5‐NPs (**11**) including a robust coordination strategy of copper ions in the fluorescent copper‐loaded nanoparticle. b) Schematic illustration for the synthetic strategy for the preparation of nanocatalyst Cu@BTTAA‐Cy5‐NPs (**11**).

Monodispersed cross‐linked polystyrene NPs were obtained by dispersion polymerization as previously described by our group.^[^ ^33^ ^]^ The NPs were then bifunctionalized using standard Fmoc solid‐phase protocols (see details in Supporting Information). Briefly, a fluorenylmethoxycarbonyl‐protected poly(ethylene glycol) (Fmoc‐PEG) spacer was conjugated to amino‐functionalized cross‐linked polystyrene NPs, as previously reported.^[^ ^29^ ^]^ Next, a lysine orthogonally protected with Fmoc and Dde (*N*‐(1‐(4,4‐dimethyl‐2,6‐dioxocyclohexylidene)ethyl)) was conjugated.^[^ ^34^ ^]^ The alpha and epsilon amino groups of this lysine residue were used to conjugate both moieties, the BTTAA ligand and the Cy5 fluorophore, through amide chemistry using oxyma/DIC as coupling reagents. BTTAA (2‐(4‐((bis((1‐(tert‐butyl)‐1H‐1,2,3‐triazol‐4‐yl)methyl)amino)methyl)‐1H‐1,2,3‐triazol‐1‐yl)acetic acid) was selected as a robust copper(I)‐stabilizing ligand known to improve the efficiency of CuAAC reactions and enable solid‐supported catalysis.^[^ ^35^, ^36^, ^37^, ^38^, ^39^ ^]^ Its synthesis is straightforward, yielding the ligand in high purity and good yield, which makes it a practical and reproducible option for surface modification (Scheme S1, Supporting Information). Importantly, BTTAA contains a carboxylic acid functional group that enables covalent coupling to amine groups on the nanoparticle surface, a critical aspect of our functionalization strategy.^[^ ^26^, ^40^ ^]^ Sulfo‐Cy5 was incorporated as a fluorescent tag to enable nanoparticle tracking. The result was NPs that were functionalized with both moieties (BTTAA‐Cy5‐NPs, **10**) prior to copper loading, which are used as controls. Metal coordination was ultimately achieved by treating the nanoparticles with excess CuBr in DMF under agitation. Following coordination, the nanoparticles were purified through sequential centrifugation steps using DMF, methanol, and deionized water to remove excess copper and uncoordinated copper species.^[^ ^25^, ^27^, ^28^, ^30^ ^]^ This purification step ensures the removal of free copper and contributes to the stability of the final nanocatalyst. This process yielded the final product, Cu@BTTAA‐Cy5‐NPs (11) (**Figure** **1**A; see Supporting Information for full details). As a control, nanoparticles functionalized only with BTTAA (Cu@BTTAA‐NPs, **7**) were synthesized (Scheme S2, Supporting Information).

**Figure 2:**
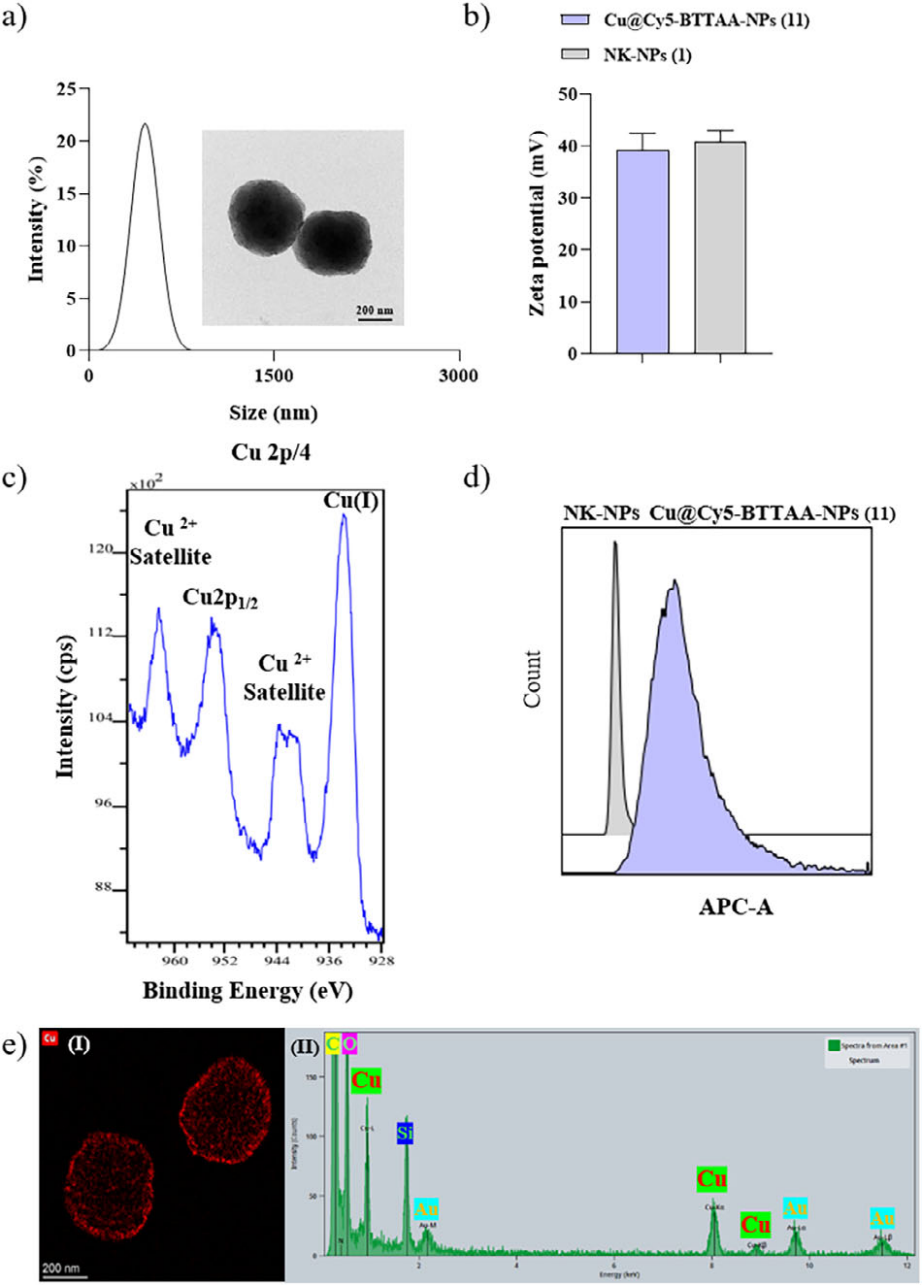
Physicochemical characterization of the Cu@BTTAA‐Cy5‐NPs (**11**). a) Hydrodynamic diameter values determined by DLS. Insets are representative TEM images; b) zeta potential values of Cu@BTTAA‐Cy5‐NPs (**11**) vs NK‐NPs **(1)**; c) XPS spectra for the determination of the oxidation state of nanoparticle‐coordinated copper; d) histograms for Cu@BTTAA‐Cy5‐NPs (**11**) vs NK‐NPs (**1**) of the Cy5 fluorophore channel measured by flow cytometry; (e) EDX‐HRTEM image and EDX analysis highlighting the copper (Cu) signal.

The physicochemical characterization of Cu@BTTAA‐Cy5‐NPs (**11**) was carried out using standard techniques. Dynamic light scattering (DLS) confirmed the monodispersity of the nanoparticles, with hydrodynamic sizes ≈480 nm (**Table** **1** and **Figure** **2**a). Neither the functionalization with BTTAA and Cy5 nor the subsequent copper coordination significantly affected their size or monodispersity. Zeta potential measurements indicated a stable positive surface charge for all functionalized nanoparticles, similar to the naked particles (Table 1 and Figure 2b). Transmission electron microscopy (TEM) and high‐resolution TEM (HRTEM) images confirmed the spherical shape of the nanoparticles (Figure 2a; Figure S1, Supporting Information). EDX‐HRTEM imaging and elemental analysis for copper showed a largely uniform distribution of copper ions throughout the Cu@BTTAA‐Cy5‐NPs (**11**) and Cu@BTTAA‐NPs (**7**) (Figure 2e; Figure S1, Supporting Information).

**Table 1:** Physicochemical characterization of Naked‐NPs, Cu@BTTAA‐NPs (**7**), and Cu@BTTAA‐Cy5‐NPs (**11**).

	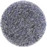	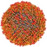	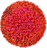
Property	NK‐NPs (1)	Cu@BTTAA‐NP (7)	Cu@BTTAA‐Cy5‐NP (11)
Hydrodynamic Size (nm)	484.6	485.3	483.5
Polydispersity Index (PDI)	0.119	0.111	0.179
ζ‐potential (mV)	+40.79	+37.61	+39.17
Shape	Spherical	Spherical	Spherical
Copper Content (ppm)	N/A	2.58	20
Copper Content (mmol/NP)	N/A	4.24E‐17	4.63E‐16
Sulfo‐Cy5 Content (nmol/NP)	^–^	^–^	7.90E‐12

A critical aspect for CuAAC catalysis is the copper oxidation state. X‐ray Photoelectron Spectroscopy (XPS) analysis of Cu@BTTAA‐Cy5‐NPs (**11**) revealed peaks corresponding to both Cu(I) (binding energy ≈932.5 eV) and Cu(II) species (binding energy ≈934.5 eV, accompanied by shake‐up satellites) within the nanoparticles (Figure 2c). This confirms that the BTTAA ligand effectively stabilizes the catalytically active Cu(I) state upon coordination with CuBr, although a significant portion of Cu(II) is also present. The presence of Cu(II)  was clearly due to the oxidation of Cu(I), which is thermodynamically instable.

Inductively Coupled Plasma Mass Spectrometry (ICP‐MS) was used to quantify the copper loading per nanoparticle. As summarized in Table 1, Cu@BTTAA‐NPs (**7**) showed a copper content of 2.58 ppm, corresponding to 4.24E‐17 mmol of Cu per NP. Strikingly, Cu@BTTAA‐Cy5‐NPs (**11**) exhibited an approximately ten‐fold higher copper loading (20 ppm, corresponding to 4.63E‐16 mmol of Cu per NP) compared to Cu@BTTAA‐NPs (**7**). This observation suggests that the sulfo‐Cy5 moiety, in addition to its tracking function, likely contributes significantly to copper coordination within the nanoparticle. Coordination of copper ions with sulfo cyanine dyes is known and can occur through ionic interactions with their sulfo groups and interactions with their π electron system.^[^ ^31^, ^32^, ^41^, ^42^, ^43^ ^]^ We thus hypothesize that the presence of the sulfo‐Cy5, potentially in concert with the cross‐linked aromatic regions of the polystyrene matrix itself, provides additional coordination sites to enhance copper loading capacity beyond that afforded by BTTAA alone. Furthermore, the collapsed spherical structure of the cross‐linked polystyrene nanoparticles may positively contribute to this effect (Figure 1a). Then, we corroborated the atomic percentage of copper by XPS showing good correlation with the total copper content measured by ICP‐MS across the different nanoparticle formulations (see Table S1, Supporting Information), supporting the highest copper content in NP **11** than in NP **7**. These results suggest a cooperative or supplementary coordination role for the dye and/or the nanoparticle matrix beyond the BTTAA ligand. Interestingly, a slight quenching of Cy5 fluorescence was observed (Figure S2, Supporting Information), which may support the presence of paramagnetic copper(II) species generated during the CuBr metalation process.

Importantly, despite the significant copper loading and potential interactions between copper and the Cy5 fluorophore, FACS (Fluorescence‐Activated Cell Sorting) analysis confirmed that the Cu@BTTAA‐Cy5‐NPs (**11**) retained their fluorescence properties, showing clear detection in the APC‐A channel (Figure 2d). This retention of fluorescence is critical, as it validates the nanoparticle's capability to be tracked, enabling the real‐time monitoring essential for our proposed in situ optimization approach. FACS analysis of control NK‐NPs (**1**) and Cu@BTTAA‐NP (**7**) showed no signal in this channel, as expected.

This comprehensive physicochemical analysis, including DLS, TEM, XPS, and ICP‐MS, confirmed the formation of monodispersed, spherical nanoparticles with significant copper loading.

### 2.2 Optimization Conditions of Copper NPs as Catalyst

Once the NPs could be prepared and characterized, we validated their performance as catalysts in CuAAC reactions in vitro. The objective was to compare the catalytic efficiency of the tracker nanocatalyst Cu@BTTAA‐Cy5‐NP (**11**) with the non‐fluorescent Cu@BTTAA‐NPs (**7**) and control NK‐NPs (**1**) using the reaction of 4‐azidoanisole (**A‐3**) and phenylacetylene (**Phe**) to form **14c** as a model (**Table** **2**). Reaction conditions were systematically screened by varying the copper concentration (immobilized on the nanoparticles), the presence or absence of a reducing agent, and the solvent composition. This study aimed to assess the catalytic performance of the NPs by examining their effect on the reaction conversion rate (%) and product yield (%). The reaction conditions included the presence or absence of ascorbic acid, a well‐known reducing agent of Cu(II) to Cu(I) used broadly in CuAACC reactions.^[^ ^44^, ^45^ ^]^ In a first stage, the optimal conditions for the model reaction with the Cu@BTTAA‐NPs (**7**) were determined, testing different copper concentrations from 0.1 ppm to 2 ppm using a methanol‐water (1:1) mixture as solvent. As shown in entry 3, with 0.1 ppm copper, there was no reaction after 48 h. However, by slightly increasing the copper concentration to 0.5 ppm (entry 4), a 100% conversion to the click product was observed after 48 h. Continuing this trend, increasing the copper concentration to 1, 1.5, and 2 ppm resulted in significantly reduced the reaction times to 24, 5, and 1 h, respectively (entries 5–7). When we repeated the conditions of entry 7 with these NPs (**7**), but without using ascorbic acid (entry 8), we found that the reaction did not occur after 24 h, highlighting the importance of using sodium ascorbate to maintain the catalytically active Cu(I) state under these in vitro conditions.

**Table 2:** Screening conditions for the CuAAC reaction between 4‐azidoanisol (**A‐3**) and phenylacetylene (**Phe**) using nanoparticle‐based copper catalysts Cu@BTTAA‐NPs **7** and Cu@BTTAA‐Cy5‐NPs **11**.

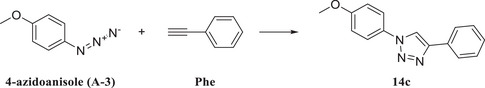								
Entry^a)^	Cu‐NPs	Cu NPs [ppm]	Cu [nmol]	Sodium Ascorbate [Asc]	Solvents	Time	Conversion^b)^ [%]	Yield^c)^ [%]
**1**	NK‐NP	–	–	YES	MeOH:H_2_O	48 h	0	–
**2**	NK‐NP	–	–	NO	MeOH:H_2_O	48 h	0	–
**3**	**7**	0.1	1.42	YES	MeOH:H_2_O	48 h	0	–
**4**	**7**	0.5	7.85	YES	MeOH:H_2_O	48 h	100	95
**5**	**7**	1	15.7	YES	MeOH:H_2_O	24 h	100	95
**6**	**7**	1.5	24.3	YES	MeOH:H_2_O	5 h	100	95
**7**	**7**	2	31.4	YES	MeOH:H_2_O	1 h	100	95
**8**	**7**	2	31.4	NO	MeOH:H_2_O	24 h	0	–
**9**	**11**	2	31.4	NO	MeOH:H_2_O	24 h	>90	80
**10**	**11**	2	31.4	NO	MeOH:H_2_O	36 h	100	95
**11** ^d)^	**11**	2	31.4	YES	MeOH:H_2_O	15 min	100	95
**12**	**11**	2	31.4	NO	MeOH	24 h	0	–
**13**	**11**	1	15.7	YES	MeOH:H_2_O	3 h	100	95
**14**	**11**	0.5	7.85	YES	MeOH:H_2_O	24 h	100	95
**15**	**11**	2	31.4	YES	H_2_O	24 h	<10	–
**16**	**11**	2	31.4	YES	H_2_O	2 h	100	95^e)^
**17**	**11**	2	31.4	YES	DMEM	3 h	100	95^e)^

^a)^ All reactions were performed at 10 µmol scale (50 mm). NPs (NK, Cu@BTTAA‐NPs **7** and Cu@BTTAA‐Cy5‐NPs **11**) were dispersed in 100 µL of solvent. 1 µmol of sodium ascorbate (0.1 eq). The reaction were done in 200 uL of mixture solvent at room temperature;

^b)^ Conversion estimated by visual TLC;

^c)^ Isolated yield;

^d)^ TON and TOF values calculated for these optimal conditions respectively 315.29 and 1261.15 h^−1^;

^e)^ 0.5 % DMSO is added to guarantee complete solubility of the reagents.

The optimal condition identified for Cu@BTTAA‐NPs (**7**) (Entry 7: 2 ppm Cu, ascorbate) was then tested with the copper‐loaded fluorescent NPs, Cu@BTTAA‐Cy5‐NPs (**11**). This yielded a significantly faster reaction, with complete conversion observed in only 15 min (entry 11), compared to 1 h for NPs (**7**). This enhanced catalytic speed with Cu@BTTAA‐Cy5‐NPs (**11**) is primarily attributed to their substantially higher copper loading capacity (Table 1), delivering more catalytic centers per nanoparticle.

We also investigated the reaction with Cu@BTTAA‐Cy5‐NPs (**11**) without sodium ascorbate. When performed in pure methanol (entry 12), no reaction was observed after 24 h. Interestingly, using a methanol‐water mixture and omitting ascorbic acid, the reaction proceeded to approximately 80 % conversion after 24 h (entry 9), and full conversion after 36 h (entry 10). The enhanced CuAAC reactivity observed upon changing the solvent is likely attributed to the nanoparticle's response to water, specifically related to its swelling or shrinking behavior in aqueous environments. In another hand, the fact that the reaction shows some progress without added reductant, could suggest that the specific coordination environment provided by the BTTAA, sulfo‐Cy5, and potentially the nanoparticle matrix in NP **11** might offer some intrinsic stabilization of the Cu(I) state or facilitate catalytic turnover even in the absence of excess external reductant, potentially making NP **11** more tolerant to varying redox conditions than NP **7**.

As expected, decreasing the amount of Cu@BTTAA‐Cy5‐NPs (**11**) from 2 ppm (entry 11) to 1 and 0.5 ppm total copper content (entries 13 and 14), resulted in longer reaction times to reach full conversion — from 15 min to 3 and 24 h, respectively. This trend is consistent with the reduced catalyst loading.

To broaden the scope of the study, additional reaction conditions were evaluated using physiologically relevant solvents. When water was used as the sole solvent (entry 15), the reaction yielded only trace amounts of the desired product after 24 h (<10% conversion), likely due to the poor solubility of the starting materials in aqueous media. To address this, 0.5% DMSO was added to guarantee complete solubility of the reagents, which significantly enhanced the reaction efficiency and led to complete conversion within 2 h (entry 16). This low DMSO concentration (<1% v/v) is within acceptable limits to avoid solvent‐related cytotoxicity during biological evaluation. To further assess the feasibility of performing the reaction under fully aqueous and cell‐compatible conditions, the transformation was carried out in Dulbecco's Modified Eagle Medium (DMEM) (entry 17). Under these conditions, complete conversion was achieved within 3 h, demonstrating the potential applicability of the reaction in biologically relevant environments. Additional solvent mixtures are presented in Table S2 (Supporting Information) as part of the extended screening set.

At this stage, we were able to compare the catalytic performance of the fluorescent tracker nanocatalyst Cu@BTTAA‐Cy5‐NPs (**11**) and the non‐fluorescent Cu@BTTAA‐NPs (**7**). The results indicate that Cu@BTTAA‐Cy5‐NPs (**11**) exhibit superior catalytic activity. Notably, these fluorescent nanoparticles catalyze the reaction efficiently using less than 1% copper.

Therefore, the Cu@BTAA‐Cy5‐NPs (**11**) are an attractive choice for applications requiring fast and efficient click reactions due to their exceptional catalytic performance. Their ability to operate with less than 1% copper and to perform efficiently in aqueous conditions makes them attractive for several biomedical applications.

This evaluation of the nanoparticles' catalytic activity in vitro in a model CuAAC reaction confirmed their efficiency. Compared to Cu@BTTAA‐NPs (**7**), the Cu@BTTAA‐Cy5‐NPs (**11**) exhibited significantly faster kinetics, primarily attributable to their higher copper loading. We demonstrated that while Cu@BTTAA‐NPs (**7**) required sodium ascorbate for activity, Cu@BTTAA‐Cy5‐NPs (**11**) showed some catalytic turnover even without this external reductant, hinting at a potentially more robust catalytic system or intrinsic Cu(I) stabilization conferred by the nanoparticle environment.

**Figure 3:**
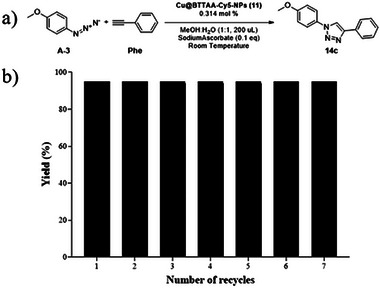
Measurement of the recyclability of Cu@BTTAA‐Cy5‐NPs (**11**) in the click reaction carried out in a MeOH:H_2_O (1:1) solvent system: a) Click reaction scheme between 4‐azidoanisole (A3) and phenylacetylene (Phe) at room temperature, 10 µmol (50 mm) scale reaction, 1 ppm of Cu@Cy5‐BTTA‐NPs (**11**), 0.1 eq of Sodium Ascorbate, 200 µL MeOH:H_2_O (1:1) and 15 min reaction. b) Yield of the CuAAC reaction using Cu@BTTAA‐Cy5‐NPs (**11**) as a catalyst over seven consecutive reaction cycles.

Finally, to test the recyclability of the Cu@BTTAA‐Cy5‐NP (**11**), the reaction of 4‐azidoanisole (**A‐3**) and phenylacetylene (**Phe**) was chosen again as the reaction model, using the conditions described in entry 11 (Table 2). At the end of each cycle (15 min), the catalyst was recovered by centrifugation, washed three times with 1 mL MeOH, and the organic layer containing the click products was concentrated, purified by flash chromatography, and analyzed by HRMS (**Figure** **3**). After seven cycles of the click reaction, the catalyst still maintained its catalytic efficiency above 95% yield. From cycle number 7, the time required for full conversion of the reaction started to increase (see Table S3, Supporting Information).

Moreover, the recyclability of Cu@BTTAA‐Cy5‐NPs (**11**), maintaining high catalytic efficiency over multiple cycles, combined with the low catalyst loading required for the reaction, suggests negligible copper leaching into the methanol–water mixtures. This was confirmed by UV–vis spectroscopy analysis (see details in Supporting Information). Several batches of triazole 14c, as well as the supernatants from the nanoparticle washing steps prior to each reuse cycle, were analyzed by UV–vis spectroscopy.^[^ ^36^, ^46^, ^47^ ^]^ Copper levels in all supernatants were below the limit of detection (LoD) (see Table S7, Supporting Information), indicating that copper contamination in the final triazole products is negligible.

Further evidence supporting the heterogeneous nature of the catalytic process was obtained through a catalyst‐filtration (hot filtration) test, commonly used to evaluate catalyst leaching.^[^ ^48^ ^]^ After removing the solid catalyst from the reaction mixture, no further product formation was observed, indicating that the reaction does not proceed in its absence (see details in Table S8, Supporting Information).

In the design and development of successful heterogeneous catalysts, the ability to recycle and reuse is a key feature that offers environmental friendliness and enhances economic viability. Therefore, based on these results, Cu@BTTAA‐Cy5‐NPs (**11**) may be a strong candidate for future development for industrial applications.

### 2.3 Scope of Applicability of Cu@BTTAA‐Cy5‐NPs (11)

To investigate the effectiveness of the Cu@BTTAA‐Cy5‐NPs (**11**) catalyst a broad click chemistry substrate scope was performed, to determine yields and reaction times achieved for various azide and alkyne combinations. Over a range of different alkyne and azide compounds, a series of reactions (**Table** **3**) were carried out using optimum conditions previously determined (Entry 11, Table 2). Briefly, a mixture of MeOH:water (1:1 ratio) as solvent at room temperature using Cu catalyst (0.314 mol%, 2 ppm), and sodium ascorbate (0.1 eq) were used. The results obtained are summarized in Table 3. All obtained products were characterized by ^1^H and ^13^C‐NMR and HRMS (see details in Supporting Information).

**Table 3:** CuAAC reactions between some different azides and alkynes using Cu@BTTAA‐Cy5‐NPs (**11**) as a catalyst.

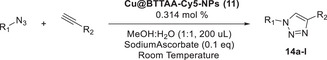					
	R_1_	R_2_	Product	t min	Yield (%)^b)^
1	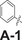	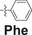	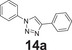	15	95
2	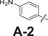	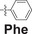	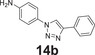	15	98
3	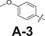	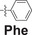	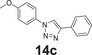	15	97
4	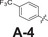	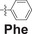	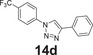	15	95
5	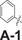	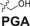	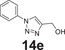	5	97
6	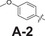	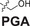	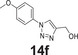	15	98
7	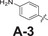	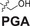	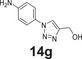	30	99
8	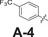	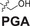	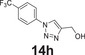	15	98
9	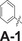	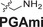	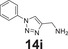	15	98
10	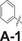	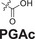	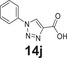	1	97
11	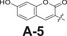	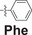	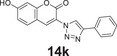	15	98
12	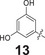	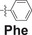	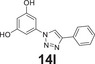	15	97

^a)^ All reactions were performed at 10 µmol scale (50 mm). Cu@BTTAA‐Cy5‐NPs (**11**) (2 ppm, 0.314 mol %) were dispersed in 100 µL of methanol. The reactions were carried in 200 µL of MeOH:H_2_O (1:1) at room temperature.

^b)^ Isolated yield. Phe: phenylacetylene, PGA: propargyl alcohol, PGAmi: Propargyl amine, PGAc: Propargyl carboxylic acid.

The results were highly encouraging, as the click reactions between azides bearing both electron‐donating or electron‐withdrawing groups and terminal alkynes with various substituents yielded the anticipated products in high yields and within short reaction times (Table 3, entries 1–11). It is noteworthy that even substrates with free amino groups, such as 4‐aminoazidobenzene or propargylamine, which typically present a greater challenge due to their tendency to form Cu(I) complexes and deactivate the catalyst, were successfully employed to produce the corresponding cycloadducts in excellent yields (entries 2, 7, and 9). Moreover, the successful application of click chemistry was demonstrated in the synthesis of fluorogenic molecules. This is exemplified by entry 11 in Table 3, in which the reactants, 3‐azido‐7‐hydroxycoumarin and phenylacetylene are not fluorescents, yet the final product **14k** (7‐hydroxy‐3‐(4‐phenyl‐1H‐1,2,3‐triazol‐1‐yl)‐coumarin) (λexc: 355 nm and λem: 449 nm). The fluorogenic reaction was used to monitor the reaction kinetic by determining the fluorescence intensity emitted at 449 nm when excited at 355 nm (See Excitation and Emission spectrum and HPLC‐FLR of compound **14k** in Figures S4 and S5 (Supporting Information) respectively in supporting information). Then, a kinetic analysis of this fluorogenic click reaction was carried out (Figure S6, Supporting Information). As the plateau phase is reached when the reagents are completely consumed, this indicates that the reaction follows first‐order kinetics with respect to this reagent as it has already been reported for CuAACC reactions.^[^ ^49^ ^]^ Finally, we tested the synthesis of a therapeutic compound that contains triazoles in its chemical structure. In particular, compound **14l** (entry 12, Table 3), a derivative of resveratrol (3,5,4′‐trihydroxystilbene; Rsv) was selected for its promising anti‐cancer potential and various beneficial properties, such as inducing cell apoptosis, lowering the risk of cardiovascular disease, and extending lifespan.^[^ ^50^, ^51^, ^52^, ^53^ ^]^ This approach would allow us to use biologically inactive alkyne and azide starting materials which, upon a click reaction, yielded a product with antiproliferative effects. This finding opened the possibility of developing cell‐based activation of bioorthogonal copper‐assisted prodrugs. Obtained products 14a‐l were characterized by ^1^H NMR, ^13^C NMR, and HRMS (see details in Supporting Information). To further evaluate the chemoselectivity and versatility of our catalytic system, we have carried out the reaction under the optimized conditions using a representative diyne substrate (octa‐1,7‐diyne). The results clearly show that only the monoclick product is formed, with no evidence of over‐functionalization (see Figure S7, Supporting Information).

Overall, high yields and short reaction times were consistently achieved across all reactions, indicating a very broad applicability of the Cu@BTTAA‐Cy5 NPs (**11**).

### 2.4 Evaluation of Efficiency of Cellular Uptake and Safety of the Nanocatalyst

Our group has previously demonstrated the remarkable versatility of cross‐linked polystyrene‐based NPs for their efficient cellular uptake across a wide range of cell lines and primary cultures and their biocompatibility in living organisms.^[^ ^27^, ^28^, ^33^, ^54^ ^]^ To demonstrate the feasibility of using these novel fluorescent copper nanocatalysts Cu@BTTAA‐Cy5‐NPs (**11**) for cellular uptake by mammalian cells in live culture, we used a difficult‐to‐target cancer cell line, the triple‐negative breast cancer cell line (MDA‐MB‐231). For this purpose, the cellular internalization of Cu@BTTAA‐Cy5‐NPs (**11**) was quantified by measuring the fluorescence intensity in the APC channel, which was correlated with the degree of cellular uptake. The results showed that cellular uptake increased significantly, as expected, with concentration of nanocatalyst (0.02–69.5 fmol Cu/cell) and time (0–24 h) (Figure S8, Supporting Information). These results demonstrating efficient cellular uptake is consistent with our previous findings regarding other metal‐loaded NPs of similar nature used as intracellular catalysts, as well as those used for intracellular barcoding.^[^ ^27^, ^28^, ^29^ ^]^

**Figure 4:**
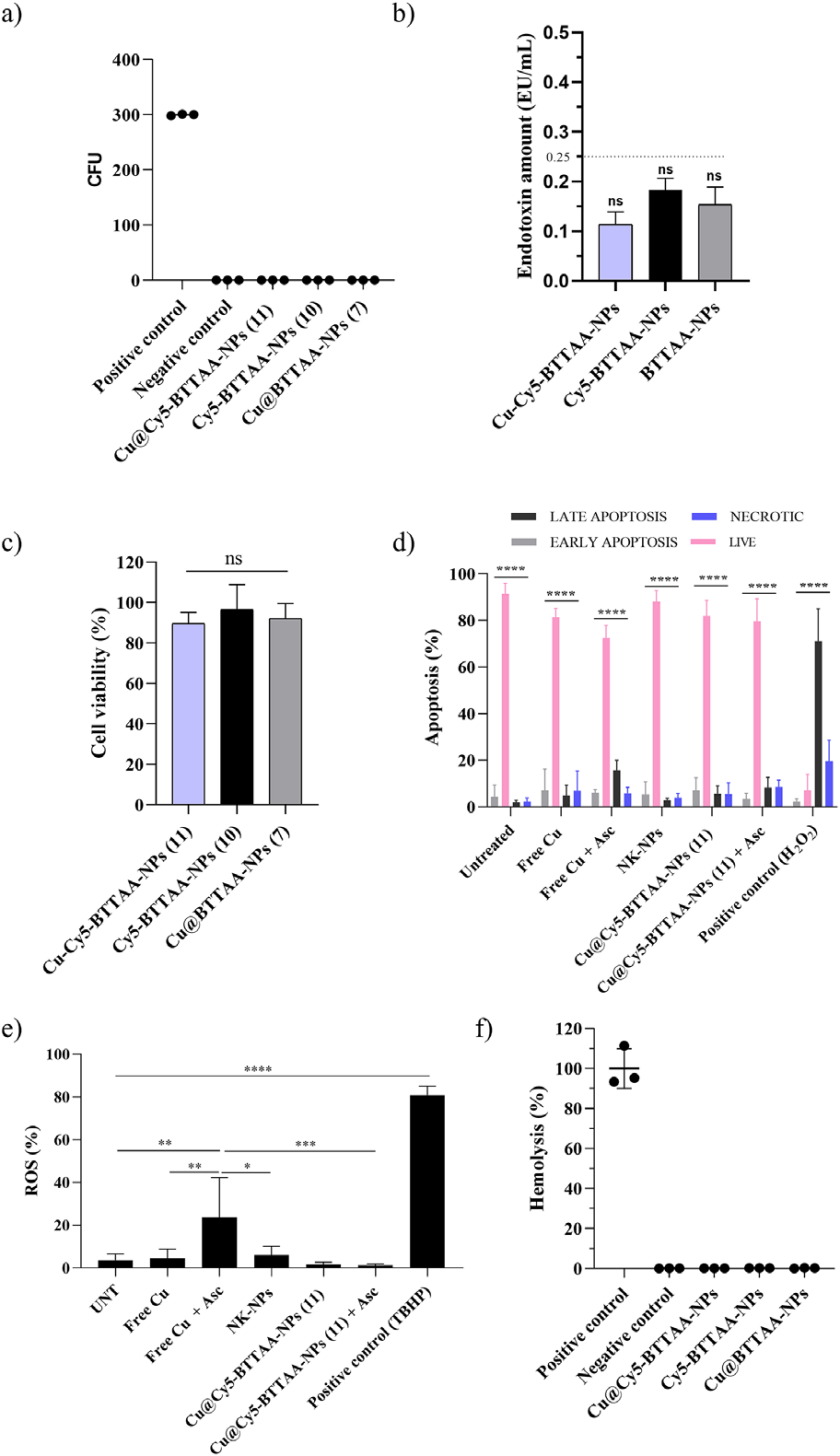
Safety evaluation of NPs. a) Scatter plot of CFU formation on agar plates after 72 h of incubation.; b) Bar graph showing endotoxin levels in the nanoparticle samples (Eu/mL); c) Viability of MDA MB 231 cell line by resazurin cell viability assay after the internalization of NPs; d) Bar graph analyses of apoptosis assessed by flow cytometry after the incubation of cells with Cu solution and NPs. Treatment with 2.5 µm of H_2_O_2_ for 4 h was used as a positive control; e) Bar graphs showing the level of ROS production by flow cytometry after the incubation of cells with Cu solution and NPs. Treatment with 400 µm of TBHP for 1 h was used as a positive control. f) Hemolytic activity of NPs in red blood cells after 3 h of incubation at 37 °C. PBS and Triton X‐100 were used as the negative and positive controls, respectively. Plotted graphs represent the mean ± SD of at least three independent experiments (*n* = 6). Statistical significance was determined by one‐way analysis of variance (ANOVA) with a Tukey's multiple comparison post‐hoc test for panels (a–f). Bars with asterisks (*) indicate the statistical difference observed between treated samples and the corresponding control groups. Two‐way ANOVA with a Tukey's multiple comparison post‐hoc test was used for panel d (** *p* < 0.0001, **p < 0.001, *: *p* < 0.01, *: *p* < 0.05).

To assess the safety and compatibility of the fluorescent copper NPs Cu@BTTAA‐Cy5‐NPs (**11**), we have carried out preliminary studies aligned with Nanotechnology Characterization Laboratory (NCL)‐recommended protocols, specifically addressing sterility, cytotoxicity, ROS activity, and hemocompatibility. Several tests were performed using NK‐NPs, BTTAA‐Cy5‐NPs (**10**), and Cu@BTTAA‐NPs (**7**) as controls. Initial assessments of bacterial contamination using agar plate tests showed no colony formation, while quantification of endotoxin levels revealed values below 0.25 EU/mL (**Figure** **4**a,b). These results confirm that the samples are free of bacteria and contain minimal endotoxin levels well within acceptable limits. The triple‐negative breast cancer cell line MDA‐MB‐231 was then used for in vitro cellular assays. The ability of the NPs to produce a cytotoxic effect on MDA‐MB‐231 cells was evaluated (MTT assay). Figure 4c shows a viability of ≈100% when the cells are incubated with nanoparticles, which show no toxicity. Moreover, these results indicated that Cu@BTTAA‐Cy5‐NPs (**11**) have good biocompatibility at the concentration up to 23.2 fmol. Cu(I) is generally more cytotoxic than Cu(II), due to its higher reactivity and ability to permeate cellular membranes. However, copper‐induced toxicity in mammalian cells typically occurs at concentrations exceeding 10 µm, depending on cell type and exposure time.^[^ ^55^, ^56^, ^57^ ^]^ In our system, the copper concentration remains below this threshold, and no cytotoxic effects were observed under the conditions tested. The potential of the nanoparticles to induce apoptosis or oxidative stress was also evaluated. In both assays, cells were treated with Cu@BTTAA‐Cy5‐NPs (**11**) (23.2 fmol copper), with and without ascorbic acid (500 µm), using control nanoparticles NK‐NPs (**1**) and a solution of CuBr at a concentration equivalent to the copper content in the copper‐loaded nanoparticles for comparison. Additionally, hydrogen peroxide (H_2_O_2_) and tert‐butyl hydroperoxide (TBHP) were used as positive controls for apoptosis and reactive oxygen species (ROS), respectively (Figure 4d). Treatment with copper in solution or control nanoparticles did not induce cell death or ROS, whereas copper in solution with sodium ascorbate increased both late‐stage apoptosis and ROS levels. Notably, this effect was not observed when cells were treated with Cu@BTTAA‐Cy5‐NPs (**11**). It is important to note that the cytotoxicity associated with sodium ascorbate was only observed in combination with CuBr for live‐cell experiments (Figure 4e). No toxicity was observed when cells were treated with sodium ascorbate alone or together with the reaction substrates (see Figure S9, Supporting Information).

Finally, to assess whether the formulated nanoparticles interact with blood components, a hemolysis assay was performed. NPs were incubated with red blood cells for 3 h at 37 °C to evaluate their hemolytic activity. Triton X‐100 was used as a positive hemolytic control. As shown in Figure 4f, none of the tested NPs induced hemolytic activity. According to the ASTM E2524‐08 standard, hemolysis is only considered significant when it exceeds 5%. In our study, all measured values were below 1%, indicating that the nanoparticles exhibit negligible hemolytic potential and are therefore considered hemocompatible under the tested conditions.

Addressing the critical need for safe and biocompatible copper catalysts for intracellular applications, all results obtained in these comprehensive nanotoxicity evaluations demonstrated that the developed Cu@BTTAA‐Cy5‐NPs (**11**) are highly biocompatible. They did not induce significant cytotoxicity, apoptosis, or oxidative stress in MDA‐MB‐231 cells at catalytically relevant concentrations, then they are hemocompatible, a notable improvement compared to free copper species.

### 2.5 Click Reactions in Cells

After confirming the catalytic activity of Cu@BTTAA‐Cy5 NPs (**11**), their ability to catalyze reactions in living cells was evaluated. To quantify the in situ catalytic performance of Cu@BTTAA‐Cy5‐NPs (**11**) for intracellular CuAAC, the formation of the fluorescent triazole product **14k** ‐ previously described in the initial experiments (Table 3, entry 11)‐ was measured by flow cytometry. For this purpose, the dual functionality of these NPs, acting as both catalyst and fluorescent tracker, allows precise localization within the cells, a unique feature offered by these nanodevices. The fluorescent triazole **14k** emits in a separate channel (Pacific blue channel, excitation/emission at 410/495 nm) from the Cy5‐labeled nanocatalyst (APC‐A channel, excitation/emission at 650/670 nm), allowing simultaneous monitoring of both the catalytic reaction and the nanoparticle distribution within cell populations, facilitating precise analysis of intracellular bioorthogonal click chemistry. To translate this chemistry to a cellular context, we conducted a systematic screening of intracellular CuAAC reaction conditions at various time points (1, 3, and 24 h).

For this purpose, this intracellular click reaction was optimized by systematically varying the amount of nanocatalyst (and thus the intracellular copper concentration), the reaction time, and the alkyne, azide, and ascorbate concentrations (**Table** **4**). This screening was performed to find the best conditions for reactions in cells, given the challenges of biocompatibility, toxicity concerns, and the complex intracellular medium. These conditions were monitored by flow cytometry (dot plots included in Table S4, Supporting Information). During the intracellular optimization of the CuAAC reaction, key parameters were systematically adjusted, in particular, different amounts of copper delivered by Cu@BTTAA‐Cy5‐NPs (**11)** from 4.63 to 69.5 fmol, azide (20–40 µm) and alkyne concentration (0.2–10 mm), ascorbate concentration (100 or 500 µm) and reactions times (1, 3, and 24 h) (Table 4). Initial experiments using low amounts of copper Cu@BTTAA‐Cy5‐NPs (**11**) to load 4.63 fmol copper in the reaction and ascorbate failed to produce detectable fluorescence, even when reaction times (1–24 h) and substrate concentrations (alkyne and azide) were increased. Subsequently, increasing the copper concentration to 23.2 fmol and ascorbate to 500 µm yielded significant fluorescence, reaching approximately 99% intensity within 3 h (Table 4 and Table S4, Supporting Information). Finally, tuning copper levels under these optimized conditions (11.6–57.9 fmol) revealed that 23.2 fmol in combination with 500 µm sodium ascorbate, and substrate concentrations of 0.2–40 mm of phenylacetylene and 40 µm of 3‐azide‐7‐hydroxycoumarin (**A‐5**) enabled robust and efficient intracellular CuAAC reactions with more than 95% of cells showing high fluorescence within 3 h. In light of these findings, 3 h was the chosen incubation period to achieve effective catalytic activity in living cells.

**Table 4:** Screening of intracellular CuAAC conditions.

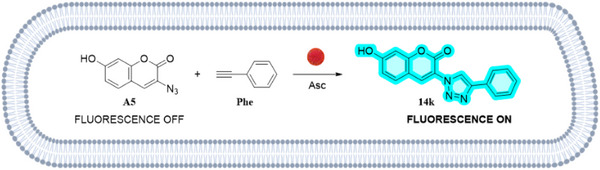								
Entry^a)^	Cu fmol	Cu % mmol	Asc µm	Phe mm	A5 µm	Time [h]	Fluorescence Intensity APC‐A channel^b)^ [count]	Fluorescence Intensity Pacific blue channel^b)^ [count]
1	4.63	0.02	100	0.2	20	3	98.3	0.12
2	4.63	0.02	100	0.2	20	24	98.3	4.48
3	4.63	0.01	100	0.4	40	24	92.9	0.08
4	23.2	0.12	500	0.2	20	3	99.8	1.66
5	23.2	0.12	500	0.2	20	24	99.6	7.13
6	23.2	0.06	500	0.4	40	1	99.2	3.65
7	23.2	0.06	500	0.4	40	3	100	33.7
8	2.32	0.01	500	5	40	3	80.9	6.68
9	4.63	0.01	500	5	40	3	90.5	18.4
10	6.95	0.02	500	5	40	3	92.0	21.5
11	9.27	0.02	500	5	40	3	94.1	34.8
12	11.6	0.03	500	5	40	3	94.4	44.5
13	23.2	0.06	500	5	40	3	99.3	93.7
14	34.8	0.09	500	5	40	3	99.6	96.1
15	46.3	0.12	500	5	40	3	99.7	97.7
16	57.9	0.15	500	5	40	3	99.9	98.5
17	69.5	0.17	500	5	40	3	99.8	98.8
18	46.3	0.12	500	1.25	40	3	99.4	10.8
19	46.3	0.12	500	2.5	40	3	99.6	34.1
20	46.3	0.12	500	10	40	3	99.4	98.7
21	11.6	0.03	500	10	40	3	98.2	94.2
22	23.2	0.06	500	10	40	3	99.2	97.9
23	34.8	0.09	500	10	40	3	99.4	98.8
24	57.9	0.15	500	10	40	3	99.2	99.1

^a)^ All reactions were performed at 0.02 nmol or 0.04 nmol scale of 3‐azido‐7‐hydroxycoumarin (A‐5) in 50 000 cells per well. Cu@BTTAA‐Cy5‐NPs 11 (4.63–57.9 fmol of Cu) were dispersed in 250 µL of DMEM and added to the cell culture for 3 h. The reactions took place inside the cells at 37 °C. Asc: Sodium Ascorbate, Phe: phenylacetylene.

^b)^ Fluorescence signals measured by FACS. Formation of derivative 14k ‐Pacific blue channel: excitation/emission at 410/495 nm and Cy5 tracker nanocatalyst‐APC‐A channel: excitation/emission at 650/670 nm.

**Figure 5:**
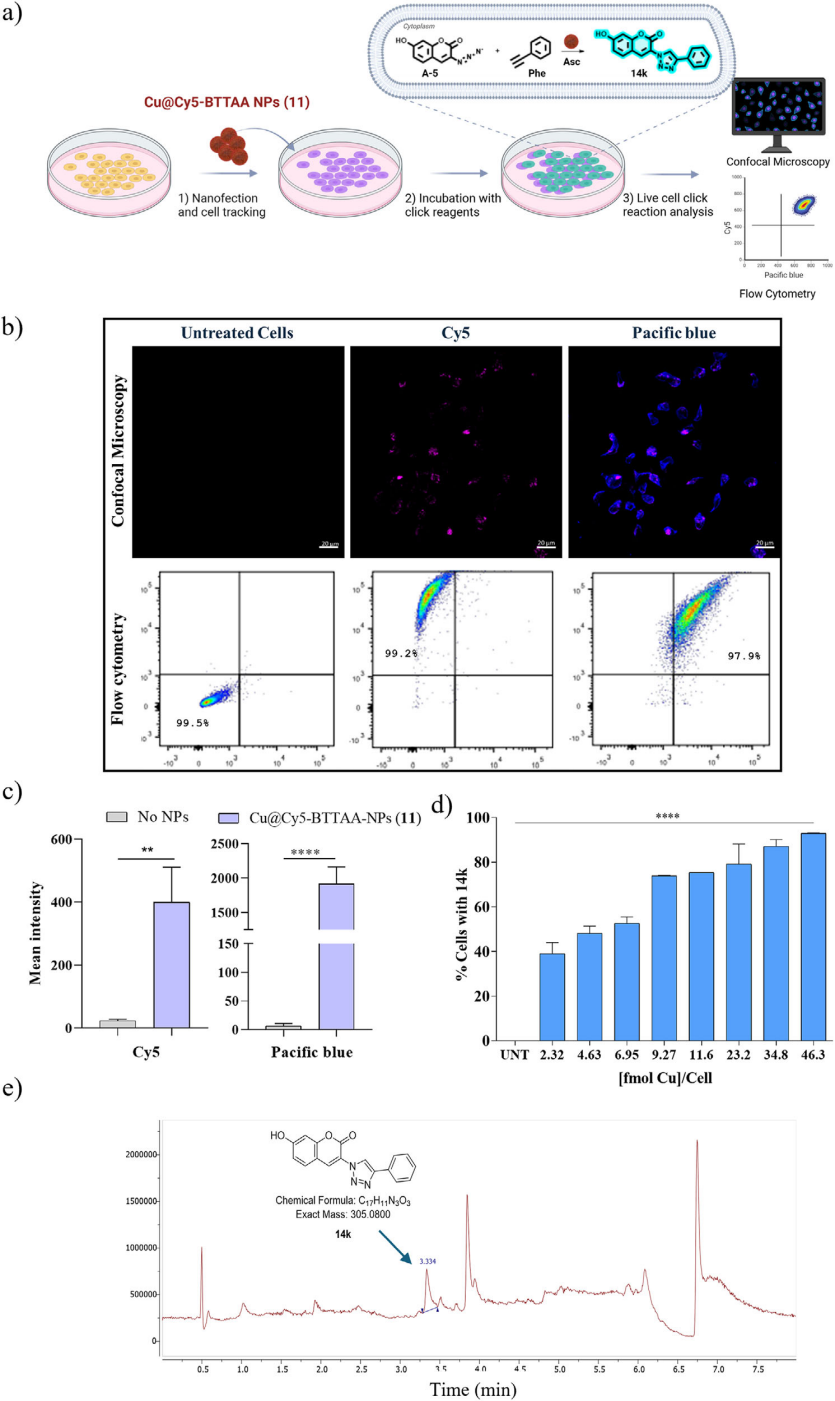
Click reactions in living cells between 3‐azido‐7‐hydroxycoumarin (**A‐5**) and phenylacetylene (**Phe**). a) Experimental protocol; b) Evaluation of the tracking ability and catalytic activity of Cu@BTTAA‐Cy5‐NPs (**11**) in MDA MB 231 cells using sodium ascorbate (Asc). Untreated cells are used as control. Reaction conditions Table 4, entry 22. Top panel: confocal microscopy. Blue for fluorescent compound and magenta for NPs **11**. The scale bar is 20 um. Bottom panel: flow cytometry. APC‐A channel shows Cu@BTTAA‐Cy5‐NPs (**11**)‐labeled cells, while the Pacific Blue channel highlights fluorescence from the intracellular click reaction. c) Comparison of fluorescence intensity in confocal images: Cu@BTTAA‐Cy5 NPs (**11**) in the APC channel and intracellular catalysis by Cu@BTTAA‐Cy5 NPs (**11**) in the Pacific Blue channel, relative to untreated cells (reaction conditions Table 4, entry 22). d) Flow cytometric analysis of the intracellular CuAAC fluorogenic response with increasing copper levels associated with Cu@BTTAA‐Cy5‐NPs (**11**). Formation of derivative 14k ‐Pacific blue channel: excitation/emission at 410/495 nm and Cy5 tracker nanocatalyst‐APC‐A channel: excitation/emission at 650/670 nm. Experiments were performed in triplicate (*n* = 3), and results are presented as the mean ± SD. For panel c, statistical significance was determined by an Unpaired *t*‐test. Bars with asterisk*s* indicate the statistical difference observed between treated samples and the corresponding control groups in each of the assays shown. For panel d, it was determined by ANOVA with a Tukey's multiple comparison post‐hoc test (*p value < 0.05, **p < 0.01, ***p value < 0.001). e) Detection of compound 14k in MDA‐MB‐231 cells (methanol extraction). UPLC chromatogram with UV detection at 254 nm, retention time: 3.334 min.

Next, to corroborate the potential of using this nanocatalyst to optimize intracellular reactions, the intracellular catalytic potential of Cu@BTTAA‐Cy5‐NPs (**11**), to mediate the click reaction between 3‐azido‐7‐hydroxycoumarin (**A‐5**) and phenylacetylene (**Phe**) in MDA‐MB‐231 cells was evaluated using the best condition selected from the previous screening (Table 4, entry 22) by confocal microscopy and flow cytometry analysis (**Figure** **5**a–c). To ensure efficient and uniform cell loading, cells were preincubated with Cu@Cy5‐BTTAA NPs (**11**) for 3 h. After washing to remove extracellular NPs, a combination of 3‐azido‐7‐hydroxycoumarin (**A‐5**) and phenylacetylene (**Phe**) with sodium ascorbate was added, followed by a 3‐h incubation (Figure 5a). Control cells were incubated with the reagents but without nanocatalyst. Confocal microscopy revealed that the fluorogenic coumarin derivative (7‐hydroxy‐3‐(4‐phenyl‐1H‐1,2,3‐triazol‐1‐yl)‐coumarin) (**14k**), the product of the click reaction, was formed exclusively in cells incubated with Cu@Cy5‐BTTAA NPs (**11**) and showed an increase in cytoplasmic fluorescence compared to controls Cells incubated with azide and alkyne compounds in the presence of ascorbic acid without nanocatalyst showed no signal in the Pacific Blue channel (Figure 5b,c). These results were corroborated by flow cytometry. Figure 5d shows the flow cytometric analysis of the intracellular CuAAC fluorogenic response with increasing copper levels associated with Cu@BTTAA‐Cy5‐NPs (**11**). Cells were incubated with just the click reagents 3‐azido‐7‐hydroxycoumarin (**A‐5**), phenylacetylene (**Phe**), both individually and mixed, or with compound **14k** in solution and Cu@BTTAA‐Cy5‐NPs (**11**) (23,2 fmol Cu) without click reagents, used as controls (Figure S10, Supporting Information). In contrast, cells incubated with the fluorescent NPs BTTAA‐Cy5‐NPs (**10**) without copper showed only fluorescence associated with the NPs, and cells directly incubated with the free fluorescent derivative **14k** showed no cellular fluorescence, but rather a high‐intensity background (Figure S11, Supporting Information). These results demonstrate the efficacy of Cu@Cy5‐BTTAA NPs (**11**) in catalyzing click reactions intracellularly.

Additionally, flow cytometry confirmed that when cells were incubated with different concentrations of Cu@BTTAA‐Cy5‐NPs (**11**) under the same conditions described previously, they were able to catalyze the reaction between azyde **A‐5** and alkyne **Phe**, resulting in increasing concentrations of the fluorogenic coumarin derivative **14k** (Figure 5d). This increase was dependent on the amount of nanocatalyst, producing an increase in fluorescence that was directly proportional to the amount of copper present, reaching up to 100% fluorescent cells. No fluorescence was observed when the cells were incubated with different reaction reagents (Tables S4 and S5, Supporting Information). While the fluorescence intensity detected in the APC channel serves as a valuable indicator of intracellular reaction progression, it should be considered that this measurement is inherently relative. Nevertheless, the observed increase in fluorescence intensity with increasing copper nanocatalyst concentration (Figure 5d) clearly demonstrates the dose‐dependent catalytic activity of Cu@BTTAA‐Cy5‐NPs (**11**) within living cells.

Furthermore, HPLC‐MS analysis of the cellular lysate confirmed the formation of the fluorogenic compound **14k** via the intracellular catalytic activity of Cu@BTTAA‐Cy5‐NPs (**11**) under previously optimized conditions (Figure 5e; Figure S12, Supporting Information).

**Figure 6:**
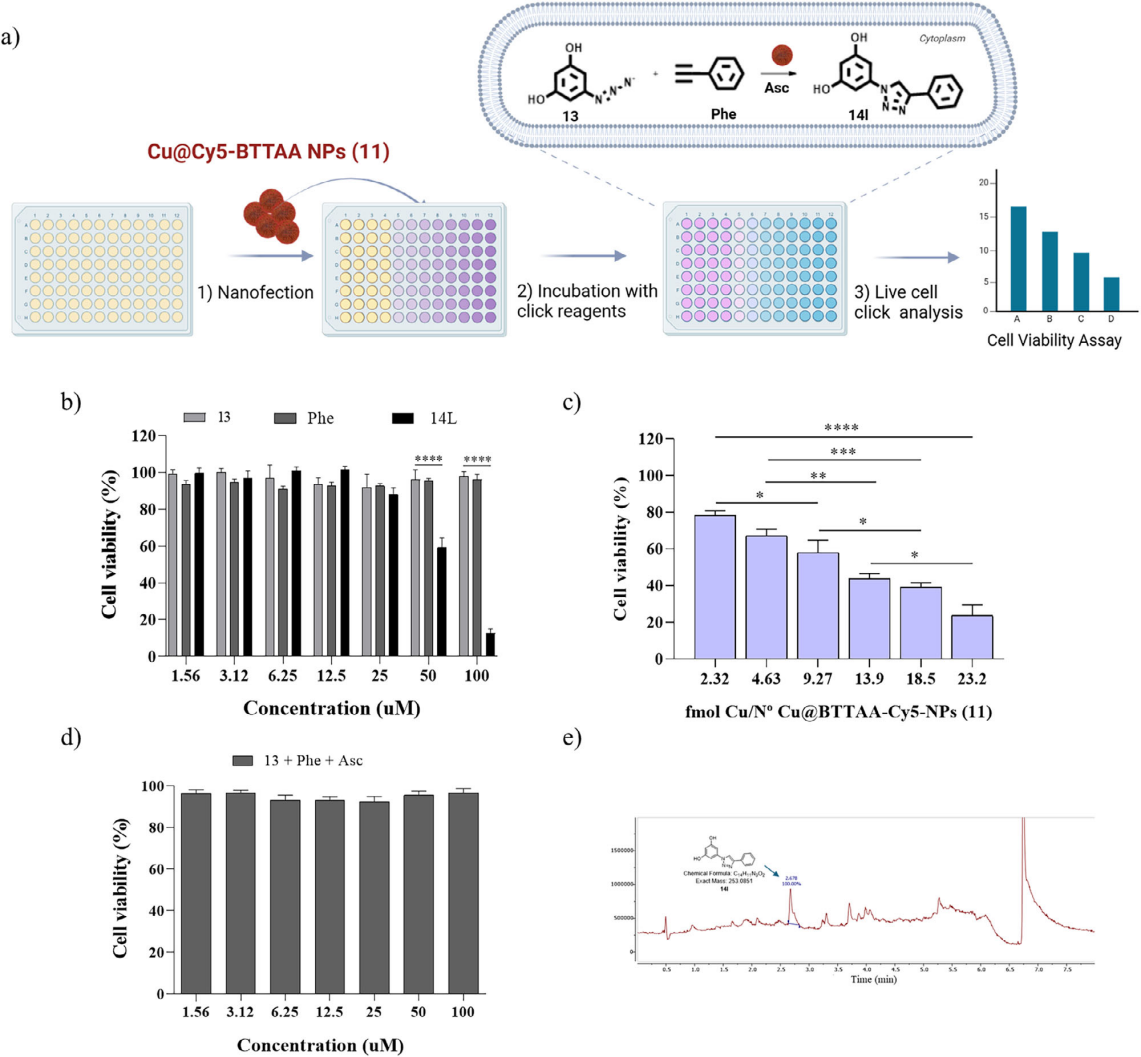
Evaluation of the cytotoxic activity of the intracellularly synthesized anticancer agent **14l**. a) Experimental protocol; b) Viability of MDA‐MB‐231 cells that were treated with free 14l compound at a range of concentrations (from 1.56 to 100 µm); c) CuAAC reaction based on Cu@BTTAA‐Cy5‐NPs (**11**) for the in situ synthesis of the chemotherapeutic drug 14l with different amounts of copper (2.32–23.2 fmol Cu); d) Viability of MDA‐MB‐231 cells treated with the precursors of reaction. Plotted graphs represent the mean ± SD of at least three independent experiments (*n* = 6). Statistical significance was determined by ANOVA with a Tukey's multiple comparison post‐hoc *test*. Bars with asterisk*s* indicate the statistical difference observed between treated samples and the corresponding control groups (**** *p* < 0.0001, ***p < 0.001, **p < 0.01, *p < 0.05). e) Detection of compound **14l** in MDA‐MB‐231 cells (methanol extraction). UPLC chromatogram with UV detection at 254 nm, retention time: 2.678 min.

Having demonstrated the efficacy of Cu@BTTAA‐Cy5‐NPs (**11**) to catalyze click reactions intracellularly, the ability of these NPs to synthesize an anticancer drug in situ using biologically inactive azide and alkyne precursors was investigated. The derivative of resveratrol previously synthesized (entry 12, Table 3) was selected for its promising anti‐cancer potential and various beneficial properties, such as inducing cell apoptosis, lowering the risk of cardiovascular disease, and extending lifespan (**Figure** **6**a). For this purpose, the solution synthesis of the triazole‐derived chemotherapeutic drug 5‐(4‐phenyl‐1H‐1,2,3‐triazol‐1‐yl)benzene‐1,3‐diol (**14l**) from 5‐azidobenzene‐1,3‐diol (**13**) and phenylacetylene (**Phe**) in the presence of Cu@BTTAA‐Cy5‐NPs (**11**) and sodium ascorbate (Asc) was successfully carried out. The cytotoxic effect was evaluated in MDA‐MB‐231 cells incubated with different concentrations of the compound **14l**, showing a significant decrease in cell viability (IC50 = 58.92 um) (Figure 6b; Figure S13, Supporting Information). These results are consistent with those previously obtained for derivatives with a similar chemical structure.^[^ ^52^ ^]^ Subsequently, MDA‐MB‐231 cells were again preincubated with different amounts of Cu@ BTTAA‐Cy5‐NPs (**11**) for 3 h, followed by the addition of a 100 µm concentration of precursor **13** and phenylacetylene (Phe) in the presence of sodium ascorbate (500 µm), and incubated for an additional 3 h. As shown in Figure 6c, there was a significant reduction in the viability of cells where in situ drug synthesis occurred in the presence of Cu@BTTAA‐Cy5‐NPs (**11**), whereas cells incubated with the CuACC reagents but without copper showed no toxicity (Figure 6d). Furthermore, HPLC‐MS analysis of the cellular lysate confirmed the formation of the compound **14l**, generated through the intracellular CuAAC reaction catalyzed by Cu@BTTAA‐Cy5‐NPs (**11**) under previously optimized conditions (Figure 6e and Figure S14, Supporting Information). Therefore, the in situ production of active drugs catalyzed by Cu@BTTAA‐Cy5‐NPs (**11**) seems to be an effective strategy to enhance cancer therapy.

## 3 Conclusion

In summary, we have successfully developed and thoroughly characterized a novel dual‐functional fluorescent copper‐loaded nanocatalyst, designated Cu@BTTAA‐Cy5‐NPs (**11**), specifically designed for controlled catalytic activity within living cells. The core achievement of this work lies in the successful translation of this system to the intracellular environment, specifically leveraging its unique dual functionality. By using the intrinsic fluorescence of the Cu@BTTAA‐Cy5‐NPs (**11**) to track catalyst localization and quantifying the formation of a fluorescent triazole product via a separate fluorescence channel, we were able to perform a comprehensive in situ screening and optimization of the intracellular CuAAC reaction conditions using fluorescence feedback. This ability to monitor catalyst presence and correlate it directly with reaction outcome within living cells represents a significant advancement, providing a powerful tool for understanding and controlling bioorthogonal chemistry in complex biological settings where bulk in vitro optimization is insufficient. The flow cytometry data explicitly demonstrated the dose‐dependent intracellular catalytic activity, confirming that the tracking capability facilitated the identification of optimal cellular reaction conditions.

Furthermore, we demonstrated the practical application of this platform by achieving the in situ synthesis of a cytotoxic triazole derivative within cancer cells from biologically inactive precursors. This proof‐of‐concept highlights the potential of using these trackable nanocatalysts for spatio‐temporally controlled prodrug activation, delivering therapeutic effect specifically where the catalyst is present and active.

Overall, the Cu@BTTAA‐Cy5‐NPs (**11**) represent a traceable nanocatalyst that enables unprecedented in situ investigation and optimization of copper‐catalyzed reactions within living cells. This work provides a valuable tool for advancing the field of intracellular bioorthogonal chemistry. While this study successfully establishes a robust proof‐of‐concept, it also lays the groundwork for several exciting future directions. The current design relies on non‐specific cellular uptake, which is effective for broad screening but could be refined by incorporating targeting ligands (e.g., antibodies or aptamers) to achieve cell‐specific delivery. Furthermore, the translation of this system from in vitro cell culture to complex in vivo models represents a compelling next step. Such studies will be essential for evaluating the nanocatalyst's biodistribution and efficacy in a physiological context, ultimately advancing its potential for targeted therapeutic applications. Future studies will focus on exploring targeted delivery strategies to extend the use of these traceable nanocatalysts for in vivo applications and other complex biological systems.

## Conflict of Interest

The authors declare no conflict of interest.

## Supporting information



Supporting Information

## Data Availability

The data that support the findings of this study are available in the supplementary material of this article.
