# Supplementary material for: Tracker Nanocatalyst for Screening of Intracellular Copper‐Catalyzed Azide‐Alkyne Cycloadditions
Source: Small. 2025 Sep 16;21(43):e06185. doi: 10.1002/smll.202506185 (PMC12571216; doi:10.1002/smll.202506185)
Supplement: Supplementary file 1 — Supporting Information [file SMLL-21-e06185-s001.pdf]

NANO · MICRO
small

Supporting Information

for *Small*, DOI 10.1002/smll.202506185

Tracker Nanocatalyst for Screening of Intracellular Copper-Catalyzed Azide-Alkyne Cycloadditions

Mónica Rodríguez-Segura, Francisco Javier López-Delgado, María Victoria Cano-Cortés, Antonio Delgado-González, Juan Jose Diaz-Mochon and Rosario Maria Sanchez-Martin**

Supporting Information

TRACKER NANOCATALYST FOR SCREENING OF INTRACELLULAR COPPER-CATALYSED AZIDE-ALKYNE CYCLOADDITIONS

Mónica Rodríguez-Segura[§,a,b,c], Francisco Javier López-Delgado[§,d], María Victoria Cano-Cortés[*,§,a,b,c], Antonio Delgado-González[a,b,c,¥], Juan José Diaz-Mochón[a,b,c] and Rosario María Sánchez-Martín[*,a,b,c]

[§]These authors have contributed equally

** Corresponding authors: María Victoria Cano Cortés and Rosario María Sánchez-Martín*

Abstract: Intracellular copper-catalyzed azide-alkyne cycloaddition (CuAAC) offers immense potential for bioorthogonal chemistry, but its application is severely hindered by copper toxicity and the challenge of controlling catalysis within the complex cellular environment. Heterogeneous copper catalysts can reduce toxicity by minimizing free copper exposure and enabling localized activity, yet optimizing their performance *in situ* within living cells remains a significant hurdle. Here, we report the development of a novel dual-functional nanocatalyst, Cu@BTTAA-Cy5-NPs, that combines robust heterogeneous CuAAC catalytic activity with intrinsic fluorescence tracking. We demonstrate the successful synthesis and characterization of these monodispersed nanoparticles, confirming efficient copper loading stabilized by BTTAA and the nanoparticle matrix, and critically, the retention of Cy5 fluorescence for tracking. This unique dual functionality allows for real-time monitoring of nanoparticle localization and correlation with catalytic product formation via distinct fluorescence channels, enabling, for the first time to our knowledge, comprehensive *in situ* screening and optimization of CuAAC reaction conditions directly within living cells using fluorescence feedback. The nanoparticles exhibit excellent biocompatibility and cellular uptake, showing no significant toxicity, apoptosis, or oxidative stress at active concentrations.

Table of content

1. Supplementary figures S1
2. Supplementary tables S8
4. Synthesis of Naked NPs (NK-NPs) S14
5. Characterization of Naked-NPs S14
5.1. Solid content (SC) of the emulsion (%) S14
5.2. Calculation of number of particles per gram S14
5.3. Calculation of loading of NPs using Fmoc NPs test S14
5.4. Qualitative ninhydrin test S15
5.5. Determination of NPs concentration (NPs/µL) by spectrophotometric method S15
6. Synthesis of BTTAA ligand (5)[2,3] S16
7. Synthesis and characterization of Cu@NPs S18
7.1. Synthesis of Cu@BTTAA-NPs (7) S18
7.2. Synthesis of Cu@Cy5-BTTAA-NPs (11) S19
7.3. Characterization of Cu@NPs S20
7.3.1. DLS and ZETA POTENCIAL S20
7.3.2. TEM Analysis S20
7.3.3. XPS S20
7.3.4. ICP-MS S20
7.3.5. EDX-HRTEM S21
7.3.6. Estimation of copper content in each NP S21
7.3.7. Detarmination of fluorophore concentration S21
8. Synthesis of Prodrug substrate 5-azidobenzene-1,3-diol (13)[5] S22
9. General procedure for the copper-NP catalyzed 1,3-dipolar cycloaddition.. S22
10. General procedure for recyling copper-NP catalyzed 1,3-dipolar cycloaddition S23
11. Determination of Copper by UV-Vis S23
12. Leaching test using hot-filtration.[9] S24
13. Relationship between volumen of Cu@BTTAA-NPs (7) and Cu@BTTAA-Cy5-NPs (11) and the concentration of copper. S25
14. Characterization of product scope Table 3 .... S26
15. NMR of compound 14l S28
16. General protocols for evaluation of intracellular activity and safety of nanocatalyst S29

16.1. Relationship between number of Cu@BTTAA-Cy5-NPs (11) and the amount of copper. S29
16.2. Cellular uptake by flow cytometry S29
16.3. Cellular uptake by confocal microscopy S30
16.4. Cell experiment quantification through LC/MS analysis S30
16.5. Cell viability S32
16.6. Safety assessment of nanoparticles S32
16.6.1. Nanoparticle bacterial contamination assessment S32
16.6.2. Endotoxin test S33
16.6.3. Apoptosis assay S33
16.6.4. ROS assay S33
16.6.5. Haemolysis Assay S34
17. Statistical Analysis S35
18. References S35

1. Supplementary figures

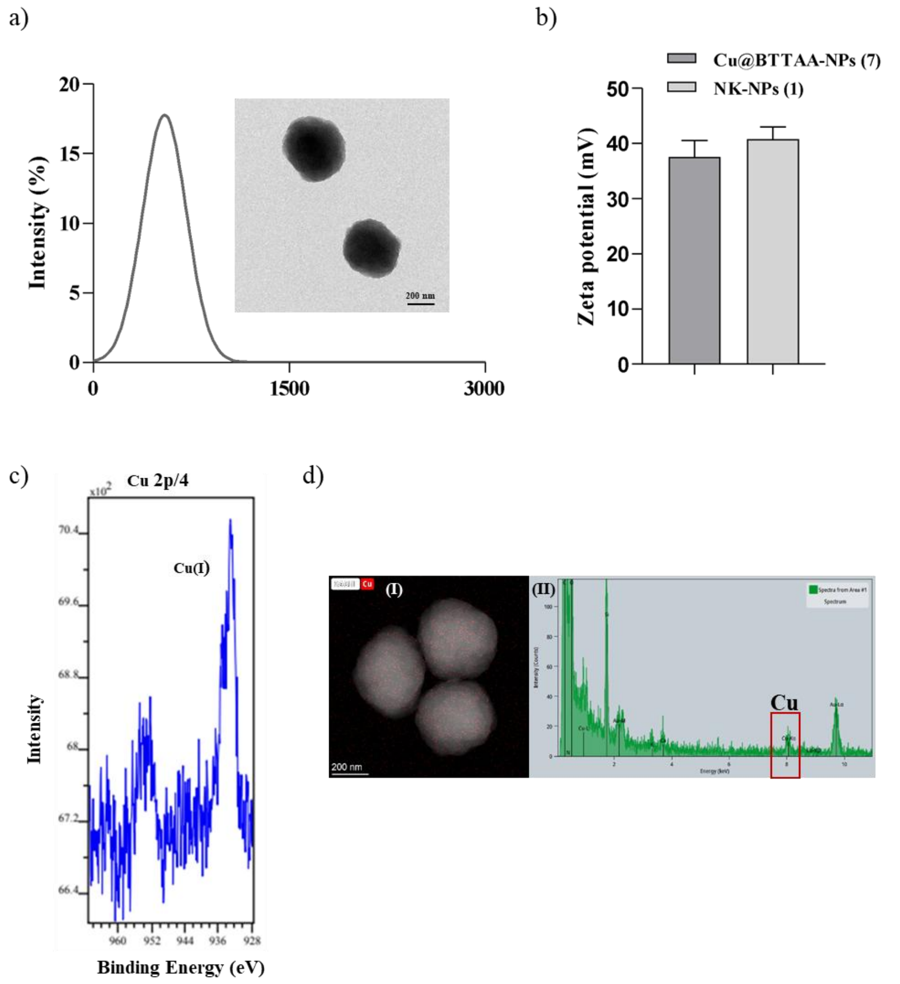

Figure S1. Physical-chemical characterisation of Cu@BTTAA-NPs (7). a) Hydrodynamic diameter values determined by DLS. Insets are representative TEM images; b) zeta potential values of **Cu@BTTAA-NPs (7)** vs. **NK-NPs**; c) XPS spectra for the determination of the oxidation state of nanoparticle-coordinated copper; d) (I) EDX-HRTEM image and (II) EDX analysis of Cu signal (copper signal in red).

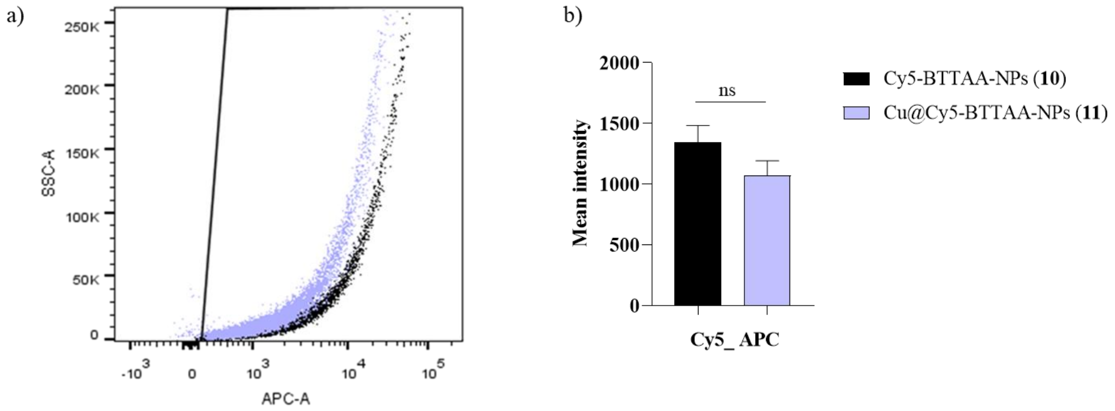

Figure S2. Comparison of the fluorescence intensity of Cu@Cy5-BTTAA-NPs (11) and Cy5-BTTAA-NPs (10). a) Flow cytometry dot plot in the Cy5 channel. b) Quantitative analysis of median fluorescence intensity, highlighting the difference between copper-loaded and copper-free nanoparticles.

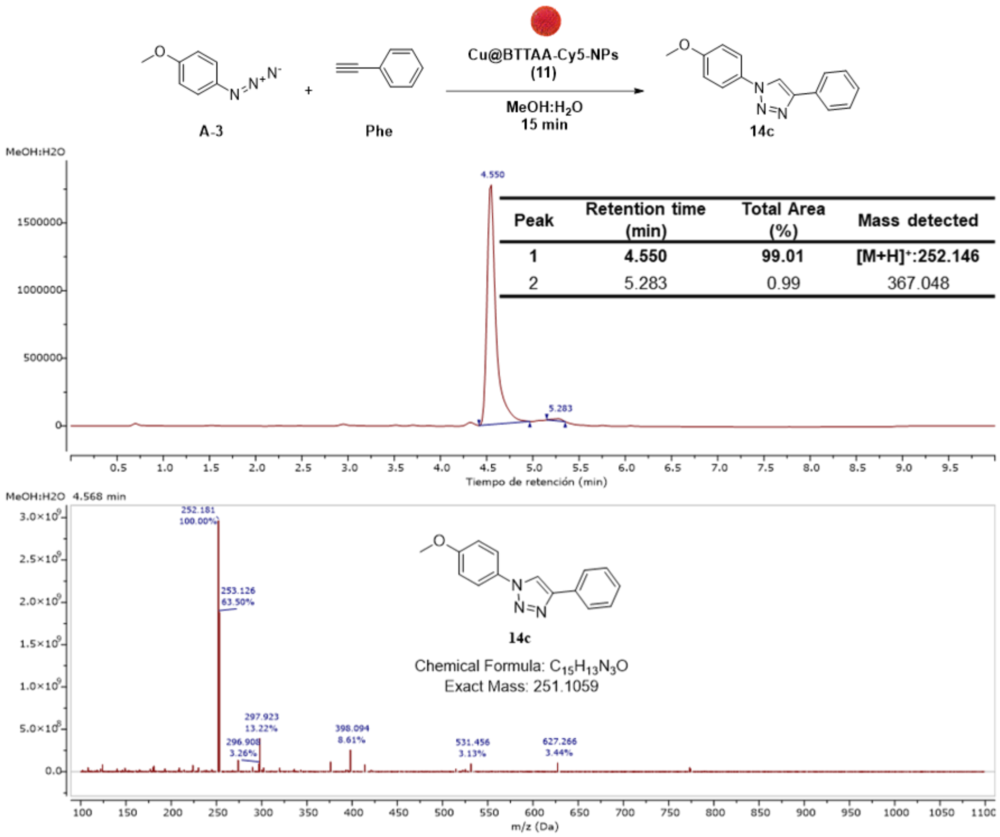

Peak	Retention time (min)	Total Area (%)	Mass detected
**1**	**4.550**	**99.01**	**[M+H]$^+$:252.146**
2	5.283	0.99	367.048

Figure S3. HPLC-UV-MS analysis of the CuAAC reaction with Cu@BTTAA-Cy5-NPs (11) in MeOH:H_2O after 15 min.

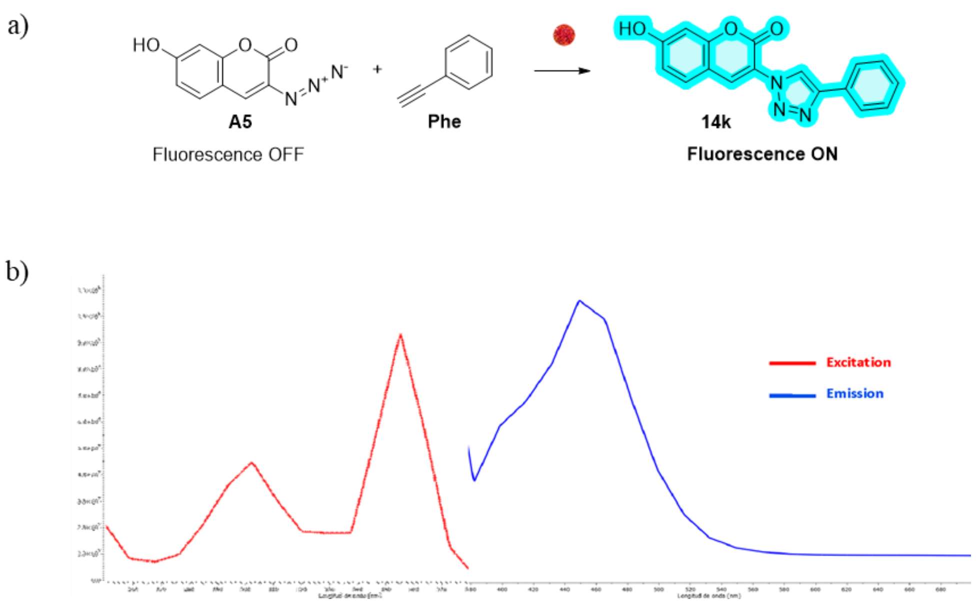

Figure S4. a) Scheme of the click reaction between 3-azido-7-hydroxycoumarin (**A-5**) and phenylacetylene (**Phe**) at room temperature to yield fluorescent compound **14k**. b) Excitation and Emission spectrum of compound 14k. Red line: Excitation spectrum (λ: 355 nm). Blue line: Emission spectrum (λ: 449 nm)

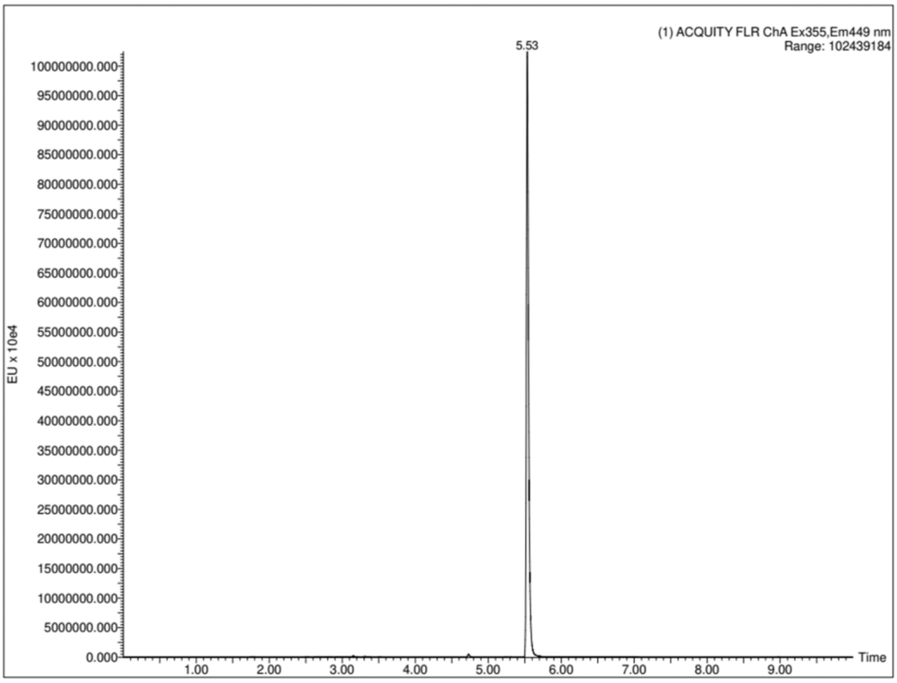

Figure S5. HPLC-FLR of compound 14k. Excitation at λ: 355 nm and Emission at λ: 449 nm.

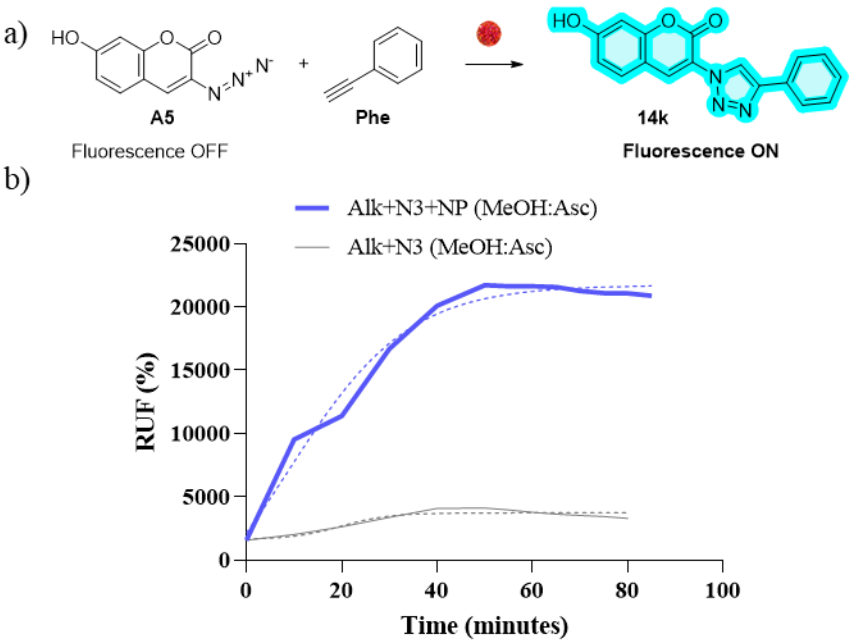

Figure S6. a) Graphical scheme of fluorogenic click reaction between 3-azido-7-coumarin (A5) and phenyl-acetylene (Phe) catalyzed with Cu@BTTAA-Cy5-NPs (11) to synthesise fluorescent compound **14k**. **b)** Kinetic analysis of the fluorogenic click reaction at 0.01 mmol scale with Cu@BTTAA-Cy5-NPs 11 (1 ppm, 0.157 mol %) (Blue line) and without Cu@BTTAA-Cy5-NPs 11 (grey line), λexc: 355 nm and λem: 449 nm

a)

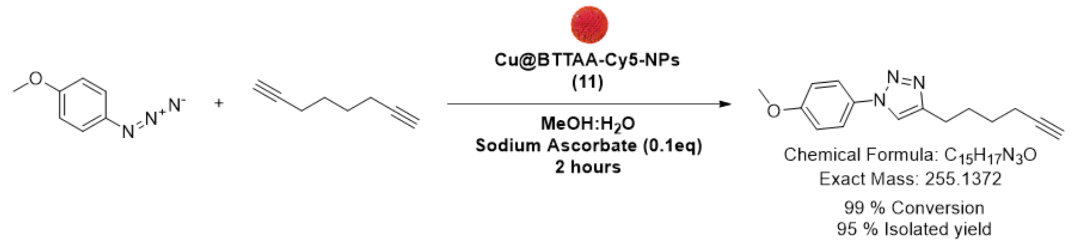

Reagent conditions: Click reaction between 4-azidoanisole (1 eq) and octa-1,7-diyne (1 eq) at room temperature, 10 μmol scale reaction, 2 ppm of Cu@BTTAA-Cy5-NPs (**11**), 0.1 eq of Sodium Ascorbate, 200 μL MeOH:H_2O (1:1) and 2 hours reaction.

b)

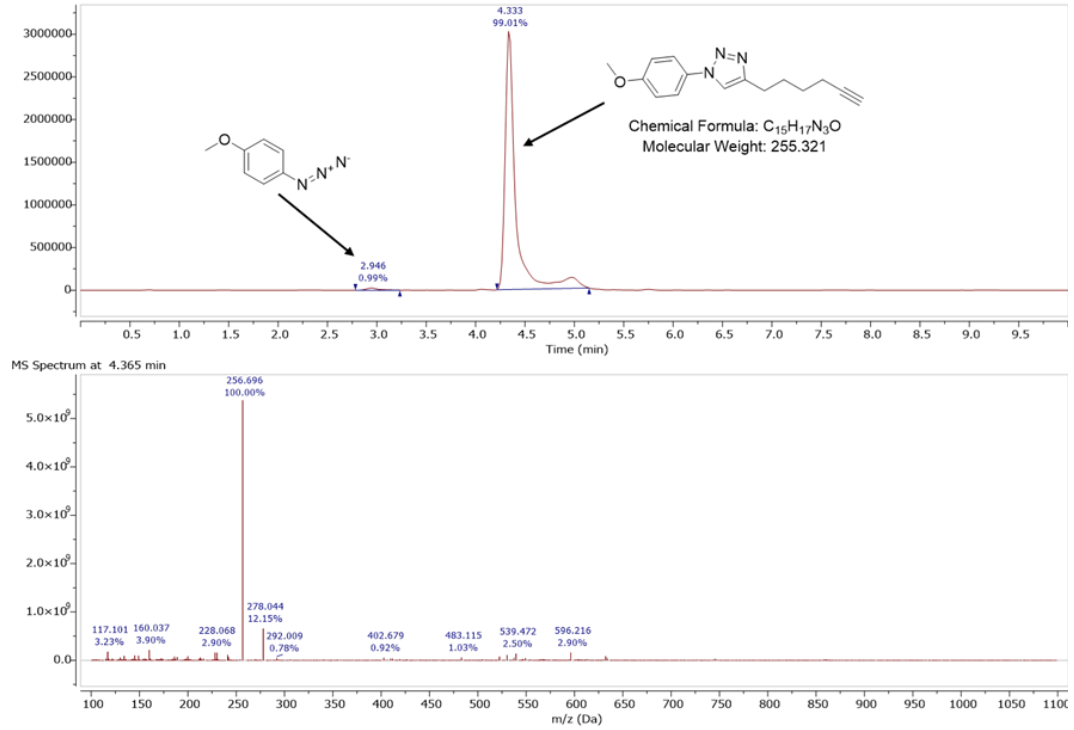

Figure S7. **Selective Mono-Click Functionalization of Octa-1,7-diyne with 4-Azidoanisole and Reaction Analysis**. a) Reaction scheme for the selective mono-click functionalization of octa-1,7-diyne and 4-azidoanisole. **Reagent conditions**: Reaction was performed at 10 μmol scale (50 mM). Cu@BTTAA-Cy5-NPs (11) (2 ppm, 0.314 mol %) were dispersed in 100 μL of methanol. The reactions were carried out in 200 μL of MeOH:H_2O (1:1) at room temperature. b) HPLC-UV-MS spectrum illustrating the reaction outcome**.**

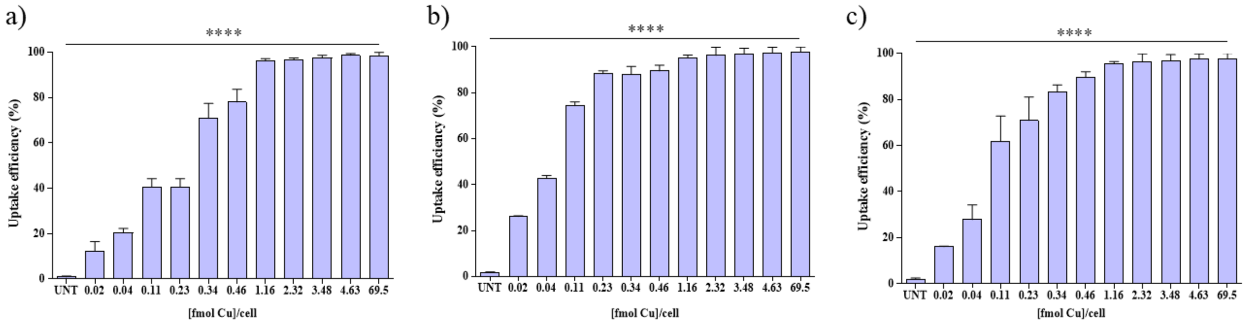

Figure S8. Evaluation of nanofection of Cu@BTTAA-Cy5-NPs (11). The MDA-MB-231 cell line was incubated with increasing concentrations of **Cu@BTTAA-Cy5-NPs (11)** for a) 3 hours, b) 6 hours and c) 24 hours. The results were analyzed using flow cytometry.

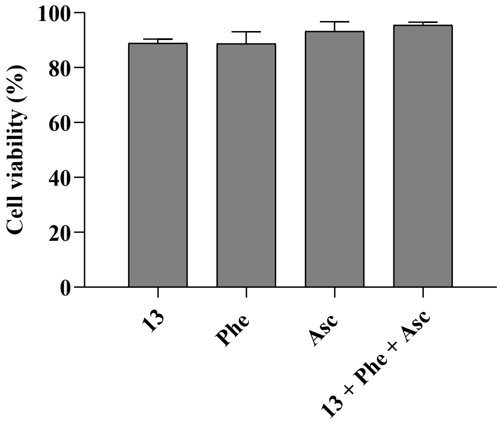

Figure S9. Viability of MDA-MB-231 cells treated with the precursors of reaction, 5-azidobenzene-1,3-diol (**13**), phenylacetylene (**Phe**) and sodium ascorbate (Asc) . Plotted graphs represent the mean ± SD of at least three independent experiments.

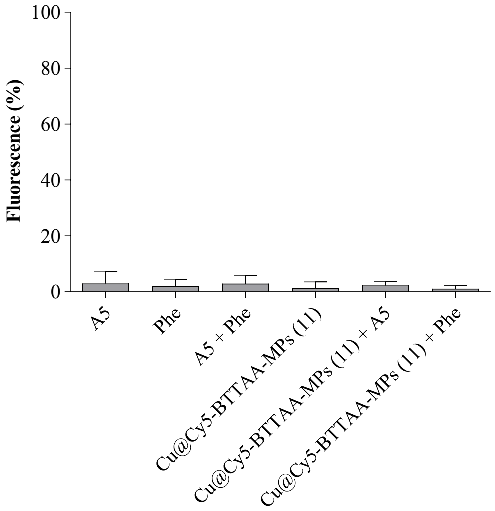

Figure S10. Controls for the validation of the catalytic ability of Cu@BTTAA NPs (11) in the click reaction by flow cytometry analysis. The MDA-MB-231 cell line was incubated with the click reagents at a known concentration corresponding to 100 µM of 3-azido-7-hydroxycoumarin (A5), phenylacetylene (Phe), and 23.2 fmol of Cu present in Cu@BTTAA-Cy5-NPs (11), either alone or with the click reagents A5 and Phe.

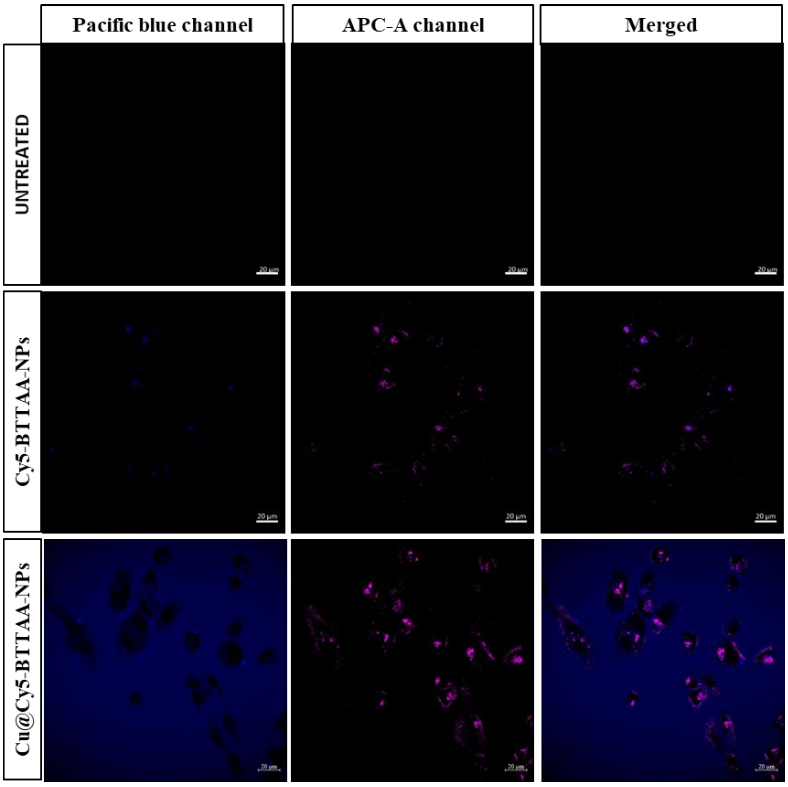

Figure S11. Confocal microscopy of Controls. APC-A channel shows **Cy5-BTTAA-NPs (10)** and **Cu@Cy5-BTTAA-NPs (11)** labeled cells, while the Pacific Blue channel highlights fluorescence from **14k** compound in solution.

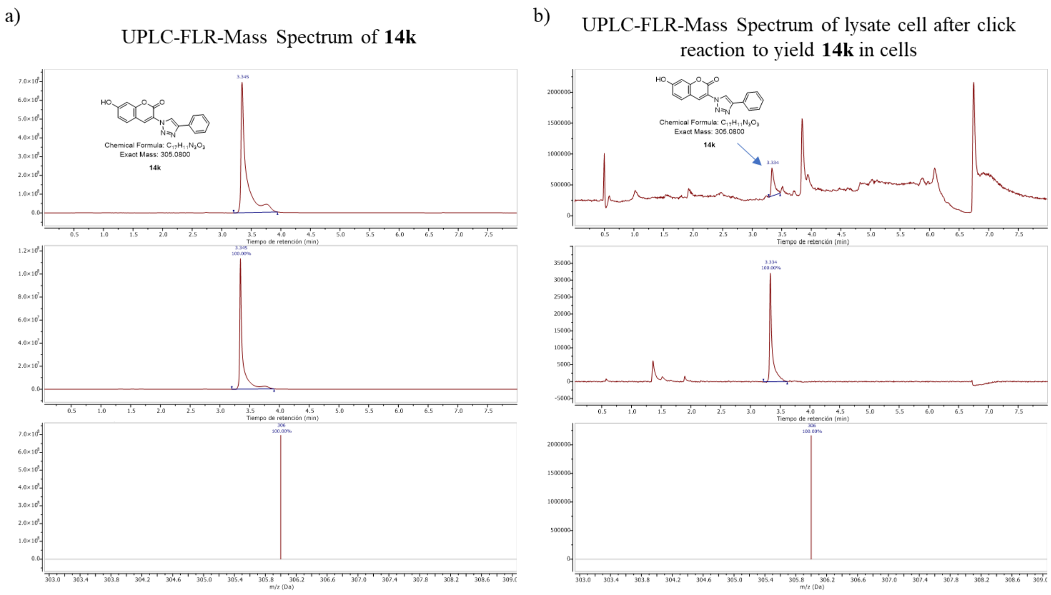

Figure S12. Detection of compound 14k pure as reference and synthesized by CuAAC intracelular with Cu@BTTAA-Cy5-NPs (11). a) Compound **14k** UPLC chromatogram with UV detection at 254 nm, retention time: 3.345min (top), with FLR detection λ_{exc}:355nm, λ_{em}:499nm (middle) and low resolution mass spectrum corresponding to the peak of 14k (bottom) **b)** Detection of compound **14k** in MDA-MB-231 cells (methanol extraction). HPLC chromatogram with UV detection at 254nm, retention time: 3.334min (top), with FLR detection λ_{exc}:355nm, λ_{em}:499nm (middle) and low resolution mass spectrum corresponding to the peak 14k (bottom).

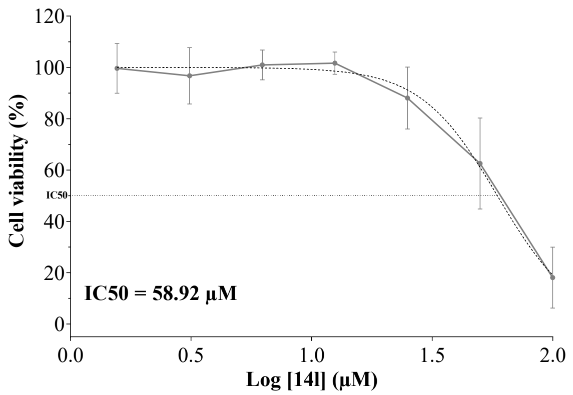

Figure S13. Dose-response curve (percentage of cell viability versus concentration) of treatment of MDA-MB-231 cancer cells with the **14l compound** formed in solution. The IC50 value was determined by the logarithm (inhibitor) versus normalized response: variable slope using the GraphPad software.

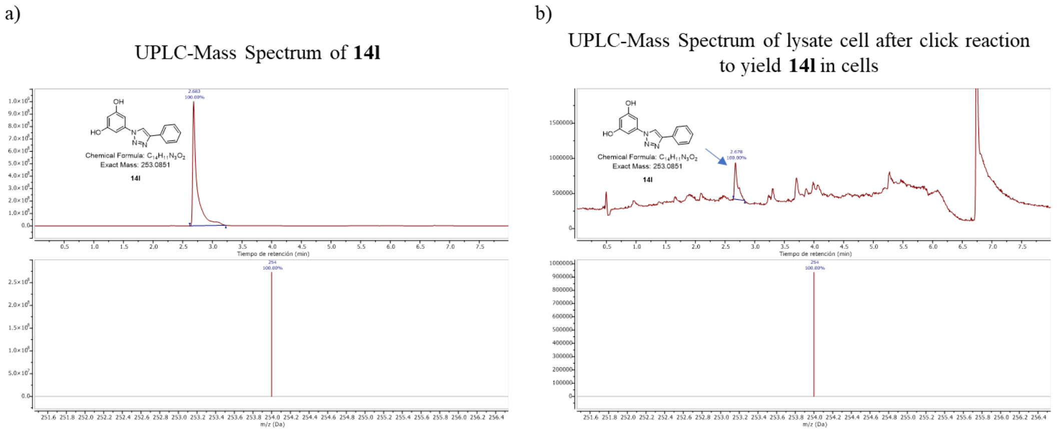

Figure S14. Detection of compound **14l** pure as reference and synthesized by CuAAC intracelular with Cu@BTTAA-Cy5-NPs (**11**). **a)** Compound **14l** UPLC chromatogram with UV detection at 254nm, retention time: 2.683min (top) and low resolution mass spectrum corresponding to the peak of **14l** (bottom). **b)** Detection of compound **14l** in MDA-MB-231 cells (methanol extraction). UPLC chromatogram with UV detection at 254nm, retention time: 2.678 min (top) and low resolution mass spectrum corresponding to the peak 14l (bottom).

2. Supplementary tables

Table S1. Relationship between XPS and ICP-MS analysis of Cu@BTTAA-NP (7) and Cu@BTTAA-Cy5-NP (11)

Cu@BTTAA-NP (7)				Cu@BTTAA-Cy5-NP (11)			
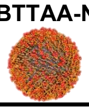				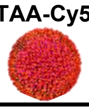			
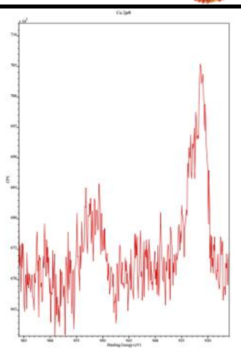				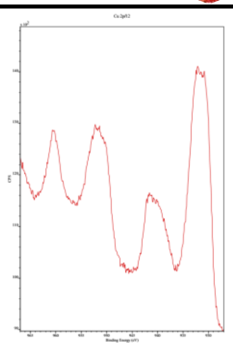			
XPS			ICP-MS (ppm)	XPS			ICP-MS (ppm)
Compounds	Position	Atomic %		Compounds	Position	Atomic %	
C	238.12	94.98		C	282.58	80.44	
N	398.41	1.29		N	397.88	4.27	
O	531.51	3.65		O	529.97	14.45	
**Cu**	**932.08**	**0.08**	**2**	**Cu**	**931.15**	**0.83**	**20**

Table S2. Complementary screening conditions for the CuAAC reaction between 4-azidoanisol (**A-3**) and phenylacetylene (**Phe**) using Cu@BTTAA-Cy5-NPs **11**.

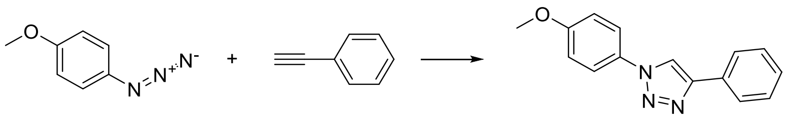

4-azidoanisole (A-3) **Phe** **14c**

Entry	Cu-NPs	Cu NPs (ppm)	Cu (nmol)	Sodium Ascorbate (Asc)	Solvents	Time	Conversion [b] (%)	Yield [c] (%)
**1**	**11**	2	31.4	YES	iPrOH:H_2O	5 h	>90	85
**2**	**11**	2	31.4	YES	tBuOH: H_2O	5 h	>90	85
**3**	**11**	2	31.4	YES	DMF: H_2O	5 h	>90	75
**4**	**11**	2	31.4	YES	DMSO: H_2O	30 min	100	95
**5**	**11**	2	31.4	YES	DMSO:DMEM	2 h	100	90

[a]10 μmol scale reaction. NPs (NK, Cu@BTTAA-NPs **7** and Cu@BTTAA-Cy5-NPs **11**) were dispersed in 100 μL of solvent. 1 μmol of sodium ascorbate (0.1 eq) The reaction were done in 200 uL of mixture solvent (1:1) at room temperature. [b] Conversion estimated by visual TLC. [c] Isolated yield.

Table S3. Recyclability of Cu@Cy5-BTTAA-NPs (11)

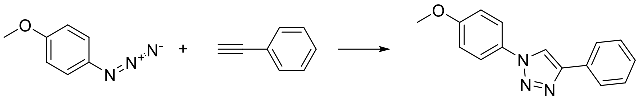

4-azidoanisole (A3) Phe 14c

Entry	Time (Minutes)	Conversion [b] (% by TLC)	Yield[c] (%)
1	15	100	95
**2**	15	100	95
**3**	15	100	95
4	15	100	95
5	15	100	95
6	15	100	95
7	30	100	95

[a]10 μmol scale reaction. **Cu@BTTAA-Cy5-NPs (11)** (2ppm, 0.314 mol %) were dispersed in 100 uL of methanol. 1 μmol of Sodium Ascorbate (0.1 eq) The reaction were done in 200 uL of MeOH:H_2O (1:1) at room temperature.
[b] Conversion estimated by visual TLC. [c] Isolated yield

Table S4. Intracellular CuAAC screening results in flow cytometry dot plots.

Entry	Fluorescence Intensity APC-A channel (dot plot)	Fluorescence Intensity Pacific blue channel (dot plot)	Entry	Fluorescence Intensity APC-A channel (dot plot)	Fluorescence Intensity Pacific blue channel (dot plot)
1	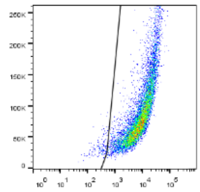	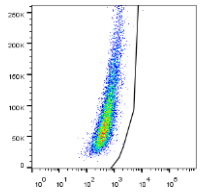	7	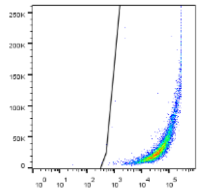	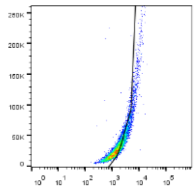
2	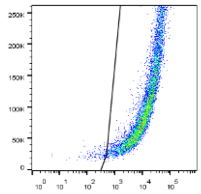	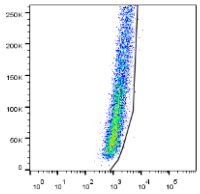	8	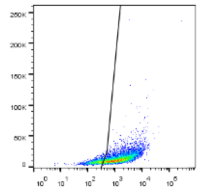	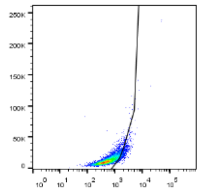
3	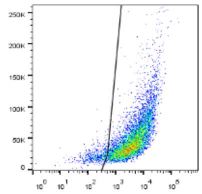	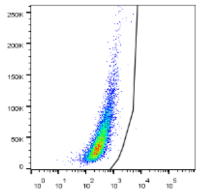	9	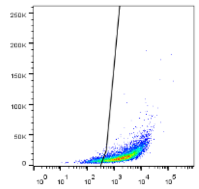	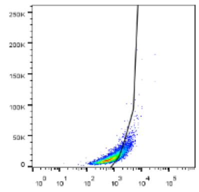

Entry	Fluorescence Intensity APC-A channel (dot plot)	Fluorescence Intensity Pacific blue channel (dot plot)	Entry	Fluorescence Intensity APC-A channel (dot plot)	Fluorescence Intensity Pacific blue channel (dot plot)
4	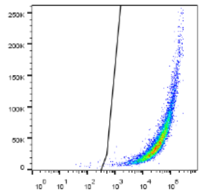	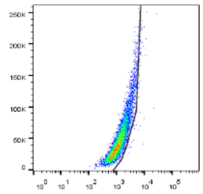	10	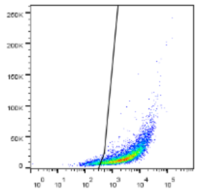	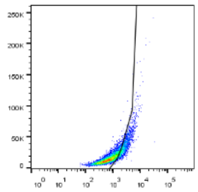
5	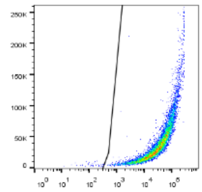	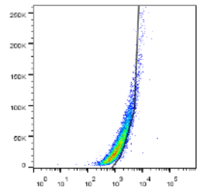	11	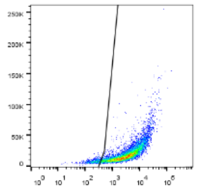	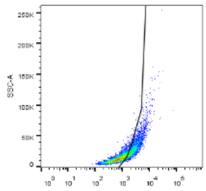
6	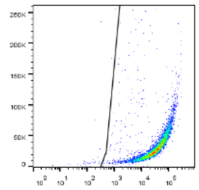	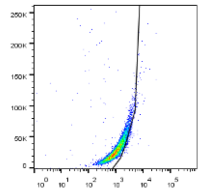	12	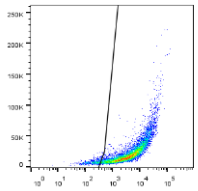	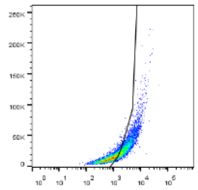
13	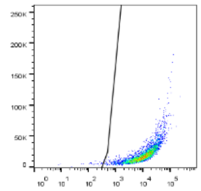	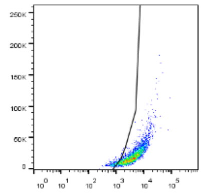	19	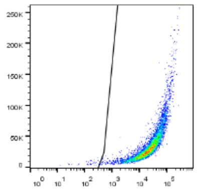	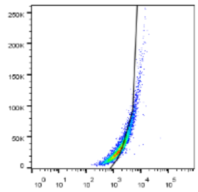
14	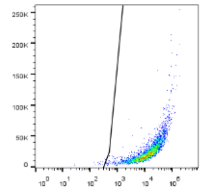	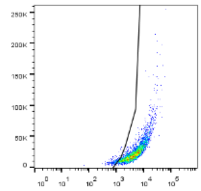	20	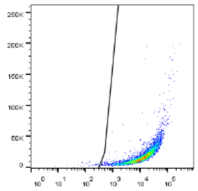	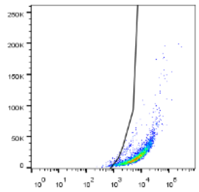
15	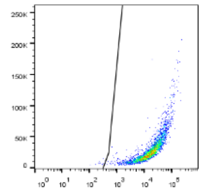	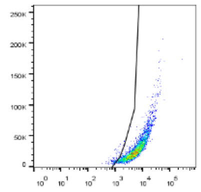	21	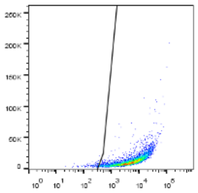	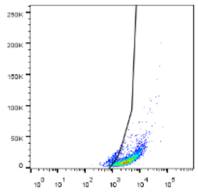
16	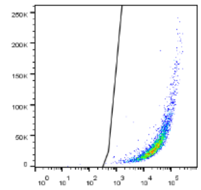	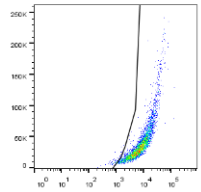	22	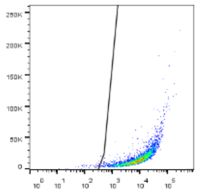	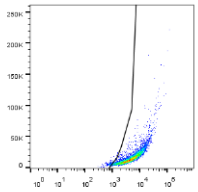

Entry	Fluorescence Intensity APC-A channel (dot plot)	Fluorescence Intensity Pacific blue channel (dot plot)	Entry	Fluorescence Intensity APC-A channel (dot plot)	Fluorescence Intensity Pacific blue channel (dot plot)
17	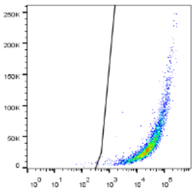	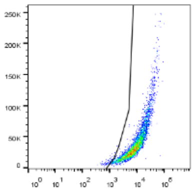	23	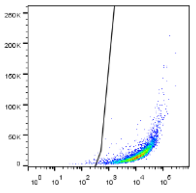	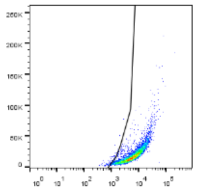
18	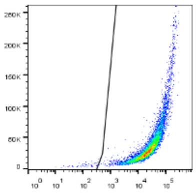	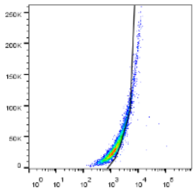	24	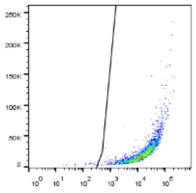	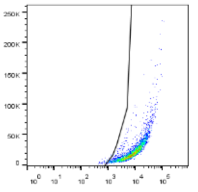

Table S5. Controls used for screening intracellular CuAAC conditions

Control	Cu fmol	Cu % mmol	Sodium Ascorbate (Asc) μM	Phenyl-acetylene (Phe) μM	A5 μM	14k μM	Fluorescence Intensity APC-A channel (count)	Fluorescence Intensity Pacific blue channel (count)
1	-	-	-	-	-	-	2.85	0.23
2	-	-	-	-	-	40	1.52	100
3	-	-	-	-	40	-	1.79	0.27
4	-	-	-	10	-	-	1.18	0.041
5	-	-	500	10	40	-	1.05	0.13
6	23.2	0.06	-	-	-	-	98.6	0.11
7	23.2	0.06	-	-	-	40	94.5	100
8	23.2	0.06	-	-	40	-	98,4	0.093
9	23.2	0.06	-	10	-	-	98.6	0.11

Table S6. Intracellular CuAAC screening results of controls in flow cytometry plots.

Control	Fluorescence Intensity APC-A channel (dot plot)	Fluorescence Intensity Pacific blue channel (dot plot)	Control	Fluorescence Intensity APC-A channel (dot plot)	Fluorescence Intensity Pacific blue channel (dot plot)
1	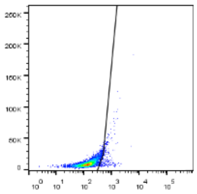	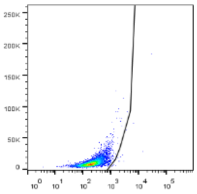	6	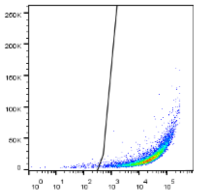	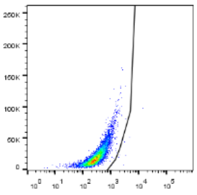

Control	Fluorescence Intensity APC-A channel (dot plot)	Fluorescence Intensity Pacific blue channel (dot plot)	Control	Fluorescence Intensity APC-A channel (dot plot)	Fluorescence Intensity Pacific blue channel (dot plot)
2	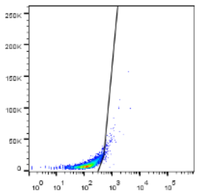	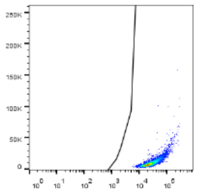	7	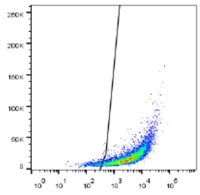	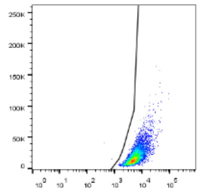
3	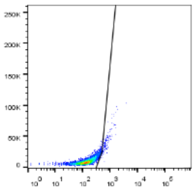	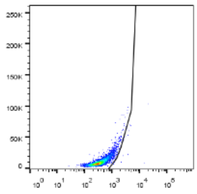	8	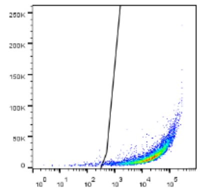	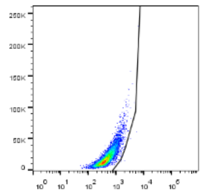
4	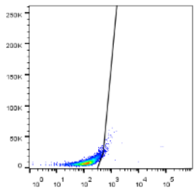	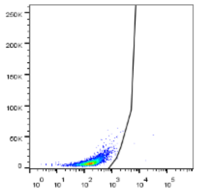	9	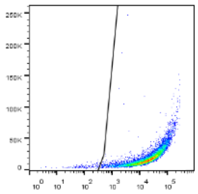	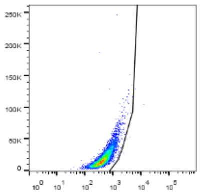
5	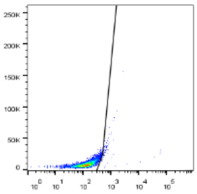	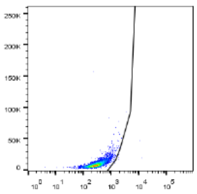			

3. Materials and methods.

All solvents and chemicals were purchased from Sigma-Aldrich, Acros and Thermo Fisher and used without further purification unless otherwise noted.

Dulbecco's modified Eagle's medium (DMEM), l-glutamine, 1% penicillin/streptomycin, trypsin-EDTA, Dulbecco's Phosphate Buffered Saline (DPBS), and fetal bovine serum (FBS) were purchased from Gibco (Thermo Fisher Scientific).

Thin-layer chromatography (TLC) was performed on Merck precoated silica gel 60 F254 aluminum sheets and vislualized by UV or $KMnO_4$ staining. Column chromatography was performed on silica gel (Merck, 230 – 400 mesh). The ^1H and ^{13}C spectra were recorded with Varian Direct Drive 400 MHz and 500 MHz spectrometers. Splitting patterns are designated as follows: s, singlet; d, doublet; t, triplet; q, quartet; m, multiplet, and br., broad. Mass spectra were recorded with a High-Resolution Mass Spectrometer LCT-TOF Premier XE, Micromass Technology and Bruker QTOF Compact. Analytical HPLC analyses were performed with an Acquity UPLC BEH™ C18, 1.7 µm, 100 x 2.1 mm column. Detection was by FLR exc: 335 nm and FLR em: 449nm. The following eluents were used: (A) H2O + 0.5% Acetic acid; (B) MeCN + 0.5% Acetic acid. HPLC grade eluents were employed, at a flow rate of 0.3 mL/min and filtered prior to injection. The following UPLC method was used: 0% to 95% B over 8 min, 95% to 0% B over 1 min. Analytical HPLC-UV-MS were performed with an Acquity UPLC BEH™ C18, 1.6 µm, 100 x 2.1 mm column at 50 ºC. Detection was by UV at 265 nm and 285 nm and mass spectra were recorded with a Low-Resolution Mass Spectrometer Xevo TQ-XS. The following eluents were used: (A) H2O + 0.1% Formic acid; (B) MeCN + 0.1% Formic acid. HPLC grade eluents were employed, at a flow rate of 0.3 mL/min and filtered prior to injection. The following UPLC method was used: 0% to 95% B over 6 min, 95% B to 100% B over 2 min, 100% B to 0% B over 0.1 min. HPLC analysis of cells lysates were also performed with an Acquity UPLC I-Class Waters, CORTECS UPLC BEH C18 1.6µm, 50x2.1 mm column at 40 ºC. The following eluents were used: (A) H2O + 0.1% Formic acid; (B) MeCN + 0.1% Formic acid. HPLC grade eluents were employed, at a flow rate of 0.4 mL/min and filtered prior to injection. The following UPLC method was used: 0% to 90% B over 6 min, 90% to 0% B over 0.1 min.

Cell cultures were performed in a NU-4750E US AutoFlow incubator from NUAIRE. Cell experiments were carried out in a laminar flow cabinet Bio II A of TELSTAR Class II A. Flow cytometry experiments were carried out on a FACSCanto II system (Becton Dickinson & Co., NJ, USA) using the Flowjo® 10 software for analysis. Confocal microscopy images were obtained using a Zeiss LSM 710 confocal laser scanning microscope and Zeiss ZEN 2010 software for image acquisition. Transmission electron microscopy (TEM) was performed on LIBRA 120 PLUS de Carl Zeiss SMT (Oberkochen, Germany). Inductively coupled plasma mass spectrometry (ICP-MS) was performed on Perkinelmer NexION 300D ICP-MS system. XPS spectra were obtained using a Kratos Axis Ultra-DLD X-ray photoelectron spectrometer equipped with an Al monochromatic X-ray source, over powdered nanoparticle samples.

4. Synthesis of Naked NPs (NK-NPs)

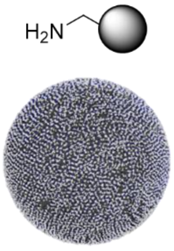

NAKED-NPs (NK-NPs)

Aminomethyl polystyrene nanoparticles (NK-NP) were synthesized via dispersion polymerization following established protocols.[1] Polyvinylpyrrolidone, PVP, (Mw 29,000, 0.05g, 1.7 µmol, Sigma-Aldrich) was dissolved in 92% ethanol/8% water for a final volume of 10 mL, and deoxygenated via argon bubbling. AIBN (7 mg, 42.4 µmol) was dissolved in styrene (freshly washed, 0.5 mL) with VBAH (7 mg, 41.3 µmol) and DVB (freshly washed, 4.65 µL). The dispersion was deoxygenated with argon bubbling before addition to the PVP/Ethanol solution. The mixture was stirred under argon for 1 hour before heating to 68 °C for 15 hours. NPs were obtained by centrifugation (11,000 G, 15 minutes) and washed with methanol (2 x 10 mL) and water (2 x 10 mL).
Finally, NPs were stored in water (10 mL) at 4°C.
Particle size distribution: mean diameter: 484.62 nm, PDI: 0.119.
Loading: 0.035 mmol / g.
Number of particles per gram: 1.73 x 10^{13}

5. Characterization of Naked-NPs

5.1. Solid content (SC) of the emulsion (%)

A known mass of a suspension of polystyrene NPs (0.5-1mg, suspended in water) was placed in a watch glass, covered with aluminium foil, dried at 25 °C for 15 hours, weighed and reweighed to give the mass of NPs. The solid content was then calculated according to the following equation:

$$\%SC = \frac{M}{Vs} \times 100$$

Where m = mass of NPs (mg), Vs = Volume of suspension (µL).

CS: 3%, 3 mg of NPs in 100 µL of solution.

5.2. Calculation of number of particles per gram

$$N = \frac{6x10^{12}}{\pi \cdot \rho \cdot d^3}$$

Where N = Number of particles/g for dry powder, ρ = Density of solid spheres (g/cm3), which is 1 g/cm^3 for polystyrene, d = diameter (nm).

Result: N= 1.73 x 10^{13} NPs per gram.

5.3. Calculation of loading of NPs using Fmoc NPs test

Fmoc-(x)-NPs (where x is Fmoc-PEG-OH or Fmoc-Lys(Dde)-OH, etc) were resuspended in 1 mL of 20% piperidine in DMF (3 x 20 min) after which the beads were washed by centrifugation three times, the supernatants combined and the loading was calculated according to the following equation:

$$Loading\left(\frac{mmol}{g}\right) = \frac{(A_{302} \cdot V)}{\varepsilon_{302} \cdot d \cdot W} x1000$$

Where A_{302}: Absorbance measured at 302 nm, V: Volume of combined supernatants (mL), ε_{302}: Molar Extinction Coefficient (7800 $M^{-1}cm^{-1}$) and W: Mass of beads (mg).

5.4. Qualitative ninhydrin test

The reaction control was determined by qualitative ninhydrin test. Small samples of NPs in methanol (6 µL, 3% SC) in a 0.5 mL capacity eppendorf were washed with methanol and centrifuged after which 6 µL of reagent A and 2 µL of reagent B were added. Mix well and heat to 100 °C for 3 min. Blue stained resin beads indicate the presence of primary amines.

5.5. Determination of NPs concentration (NPs/µL) by spectrophotometric method

The concentration of Naked-NPs (NPs per microliter) was quantified using a spectrophotometric approach as previously described. In summary, turbidity was assessed by measuring the optical density at 600 nm (OD600) of polystyrene NP suspensions using nephelometric principles. When light passes through NP suspensions, it is scattered by reflection, refraction and diffraction, with the intensity of the scattered light being directly proportional to the number of NPs in the suspension. This scattered intensity is recorded using standard spectrophotometers. Using this method, standard calibration curves were generated for 440 nm aminomethyl cross-linked polystyrene NPs based on known concentrations. These calibration curves were fitted to linear regression models which allowed the determination of the number of NPs per microliter corresponding to one unit of OD600 for each size. Consequently, these initial calibration curves allowed the estimation of NP concentrations in final batches, even after extensive handling, by measuring the OD600 of a 1 µL sample. (**Figure S15**).

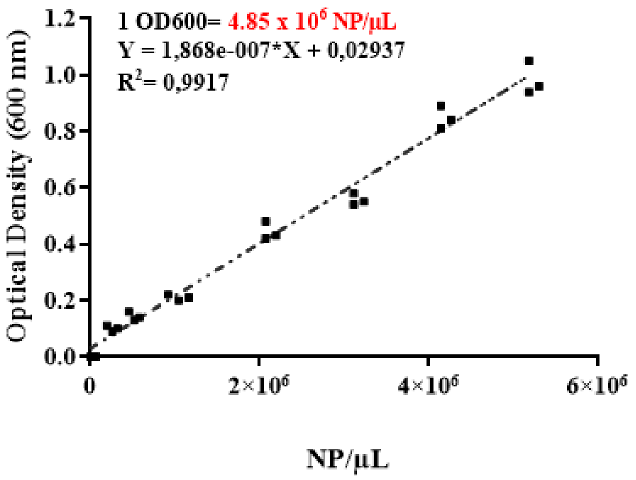

Figure S15. Calibration standard curve of concentration of Naked-NPs (OD 600)

6. Synthesis of BTTAA ligand (5)[2,3]

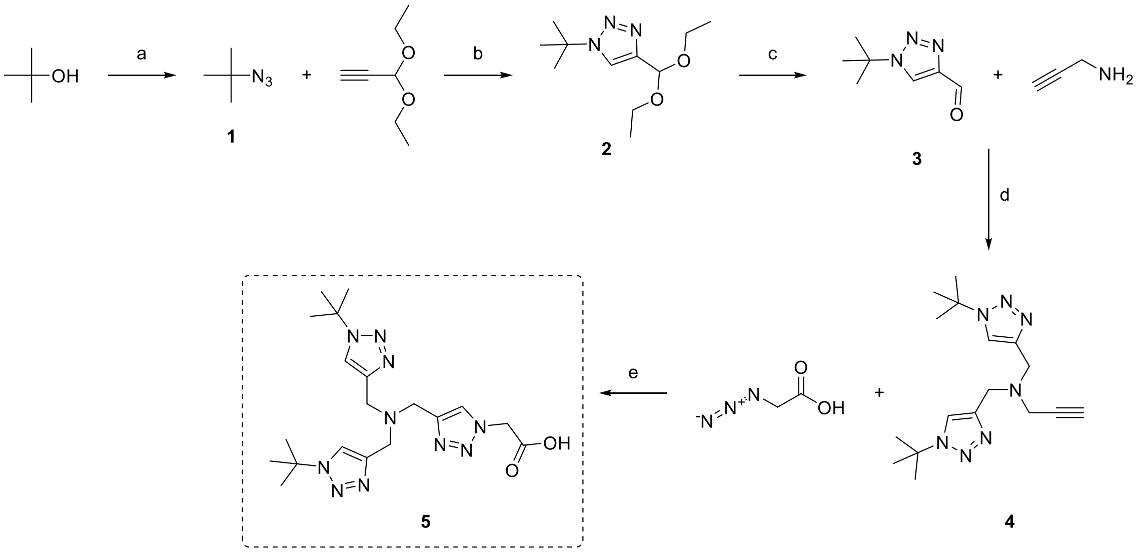

Scheme S 1. Synthesis of BTTAA ligand. Reagent and conditions: a) H_2SO_4 solution, NaN_3, *t*-butyl alcohol, 0ºC to room temperature; b) *t*-Buty-alcohol:water (1:1), $NaHCO_3$, $CuSO_4$, Sodium Ascorbate, room temperature, overnight; c) Trifluoroacetic acid, DCM, water, argon atmosphere, room temperature, 3 hours; d) Ttriacetoxyborohydride, DCM, room temperature, 40 hours; e) THF, DIPEA, tris(triphenylphosphine)copper(I) bromide, argon athmosphere, 60 ºC, overnight.

Synthesis of 2-azido-2-methylpropane (1)

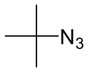

1

A solution of H_2SO_4 (55 g, 0.56 mmol, 5.6 eq.) in 55 g H_2O was prepared by the addition of 55 g H_2SO_4 to 55 g H_2O over 10 min, with vigorously stirring in an ice cooled 250 mL flask. Then sodium azide (7.2 g. 0.11 mol) was slowly added over 10 min (maintaining the temperature = 20°C in order to preclude accidental volatilization of HN_3). When all of the NaN_3 has dissolved, *t*-butyl alcohol (7.4 g. 0.1 mol) was added, and the resulting solution was stirred for 5 min and allowed to stand at room temperature for 24 h. t-Butyl azide floated to the top of the acid mixture was collected in a separatory funnel, washed with 50 ml of 2 M NaOH to remove all traces of HN_3, dried over Na_2SO_4, and clear liquid was obtained as the product. The product evaporates easily. **1H NMR (400 MHz, $CDCl_3$)** δ 1.29 (s, 9H). **13C NMR (101 MHz, $CDCl_3$)** δ 77.21.

Synthesis of 1-tert-butyl-4-(diethoxymethyl)-1H-1,2,3-triazole (2)

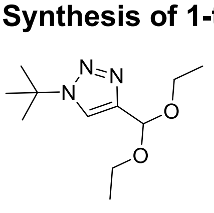

2

To a 25-mL round bottle flask equipped with a stirring bar were added 3,3-diethoxy-1-propyne (3.0 mL, 0.021 mol, 1.000 eq) and tert-butyl azide (2.394 g, 0.024 mol, 1.150 eq) in 10 mL 1:1 mixture of *t*-butyl alcohol and water. Sodium bicarbonate (2.469 g, 0.029 mol, 1.400 eq), copper(II) sulfate (0.168 g, 0.001 mol, 0.050 eq) and sodium ascorbate (0.832 g, 0.004 mol, 0.200 eq) were added to the mixture. The reaction was stirred vigorously overnight, and TLC analysis indicated the formation of a new product (Rf = 0.7 in ethyl acetate, KMnO4 stain). EDTA (2 mL, 0.5 M, pH = 8) was added, the reaction mixture was diluted with EtOAc (90 mL), washed with sat aq NaHCO3 (2 x~ 50 mL), water (2 x ~ 10 mL), and brine (30 mL). The combined organic phases were dried over anhydrous MgSO4, filtered, and concentrated in vacuo to provide 4.2 g of a light-yellow oil (4.200 g, 0.018 mol, 88.0%, 1-tert-butyl-4-(diethoxymethyl)-1H-1,2,3-triazole). The crude product was used without further purification. **^{1}H NMR (400 MHz, $CDCl_3$)** δ 7.64 (s, 1H), 5.70 (s, 1H), 3.73 – 3.57 (m, 4H), 1.66 (s, 9H), 1.24 (t, J = 7.1 Hz,

6H). **^{13}C NMR (101 MHz, $CDCl_3$)** δ 146.68, 119.04, 97.30, 61.97, 59.53, 30.13, 15.29. **HRMS calcd for [M+H]$^+$**: 228.1712, **Found** 228.1716.

Synthesis of 1-tert-butyl-1H-1,2,3-triazole-4-carbaldehyde (3)

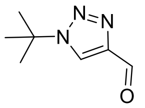

3

To a 50mL round bottom flask was added a solution of 1-tert-butyl-4-(diethoxymethyl)-1H-1,2,3-triazole (1.28 g, 5.63 mmol) in dichloromethane (6.0 mL), followed by addition of water (3.0 mL) and trifluoroacetic acid (1.0 mL). The reaction was stirred vigorously under argon for 3 h until TLC analysis indicated the complete disappearance of the starting material (10% EtOAc in dichloromethane, starting material Rf 0.5, product Rf 0.6, KMnO4 stain). The reaction mixture was diluted with EtOAc (100 mL), washed with sat aq NaHCO3 (3 × 40 mL) and brine (40 mL). The combined organic phases were dried over anhydrous MgSO4, filtered, and concentrated in vacuo to provide 0.71 g of a light yellow oil (yield: 82%). The crude product was used without purification. **^{1}H NMR (400 MHz, $CDCl_3$)** δ 10.14 (s, 1H), 8.17 (s, 1H), 1.71 (s, 10H). **^{13}C NMR (101 MHz, $CDCl_3$)** δ 185.62, 147.46, 122.63, 60.65, 30.02

Synthesis of bis[(1-tert-butyl-1H-1,2,3-triazol-4-yl)methyl](prop-2-yn-1-yl)amine (4)

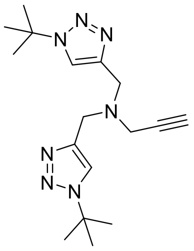

4

To a 250-mL round bottom flask was added a solution of 1-tert-butyl-1H-1,2,3-triazole-4-carbaldehyde (2.47 g, 16.1 mmol, 2.2 eq) in dichloroethane (84 mL, ~0.2 M), followed by addition of propargyl amine (361 mg, 7.2 mmol, 1.0 eq). To this mixture sodium triacetoxyborohydride (3.8 g, 17.9 mmol, 2.5 eq) was added in one portion with vigorous stirring. The reaction mixture was stirred at room temperature for 40 h. 1N H_2SO_4 (86 mL) was added to the reaction, and the mixture was stirred for 15 min. The pH was adjusted to >10 by addition of potassium carbonate. The reaction mixture was diluted with water (100 mL) and extracted with dichloromethane (3 × 300 mL). The organic layers were combined, dried over anhydrous MgSO4, filtered, and concentrated in vacuo to provide a crude product. Further purification by flash chromatography (100 g silica gel, 20% hexanes in EtOAc, Rf 0.2, KMnO4 stain) provided 1.98 g of product (yield: 84%) as a white powder. **1H NMR (400 MHz, $CDCl_3$)** δ 7.67 (s, 2H), 3.87 (s, 4H), 3.40 (d, J = 2.5 Hz, 2H), 2.28 (t, J = 2.4 Hz, 1H), 1.66 (s, 18H). **13C NMR (101 MHz, $CDCl_3$)** δ 143.67, 120.44, 77.36, 73.67, 59.35, 47.97, 42.38, 30.16. **HRMS calcd for [M+H]$^+$**: 330.2406, **Found**: 330.2408

Synthesis of 2-[4-({bis[(1-tert-butyl-1H-1,2,3-triazol-4-yl)methyl]amino}methyl)-1H-1,2,3-triazol-1-yl]acetic acid (5) or BTTAA[2]

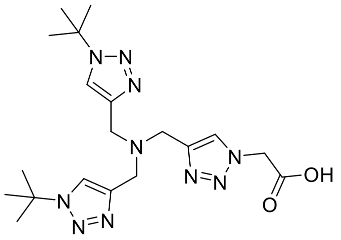

5

To a 100-mL round-bottom flask were added N,N-bis((1-tert-butyl-1H-1, 2,3- triazol-4-yl)methyl)prop-2-yn-1-amine (200.0 mg, 0.607 mmol, 1.000 eq) and 2-azidoacetic acid (88 µL, 0.914 mmol, 1.506 eq) (0.45 g, 0.45 mmol, 1.5 eq) in THF (6.0 mL). To the mixture were added N,N'-diisopropylethylamine (169 µL, 0.971 mmol, 1.600 eq) (0.62 g, 0.48 mmol, 1.6 eq) and tris(triphenylphosphine)copper(I) bromide (56.5 mg, 0.061 mmol, 0.100 eq) (0.28 g, 0.03 mmol, 10 mol%). The reaction mixture was stirred vigorously at 60 °C overnight under

argon. The crude product was concentrated in vacuo and was purified by flash chromatography (80 g silica gel, 40% MeOH in EtOAc with 1% acetic acid, Rf 0.2, $KMnO_4$ stain) to get BTTAA ligand (210.0 mg, 0.488 mmol, 80.4%) as a white solid. **^{1}H NMR (400 MHz, D_2O)** δ 8.33 (s, 2H), 8.27 (s, 1H), 5.15 (s, 2H), 4.61 (s, 6H), 1.70 (s, 18H). **^{13}C NMR (101 MHz, D2O)** δ 172.60, 136.07, 128.63, 125.09, 61.03, 47.18, 28.89. **HRMS Calculated for $[M+H]^+$**: 431.2631, **Found:** 431.2600

7. Synthesis and characterization of Cu@NPs

7.1. Synthesis of Cu@BTTAA-NPs (7)

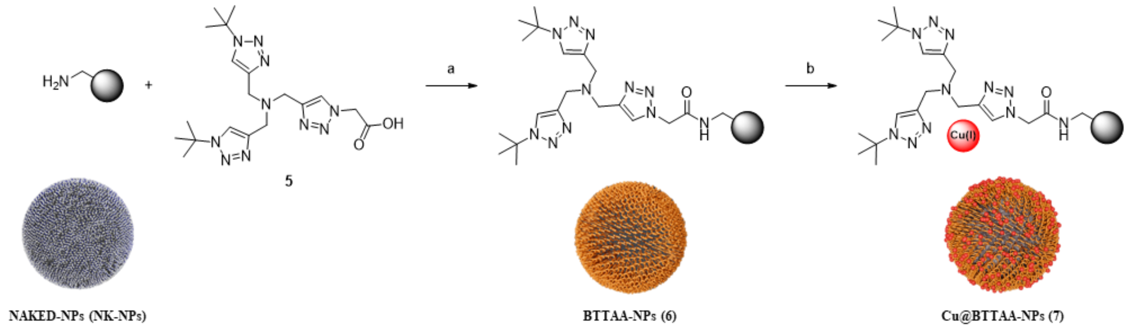

Scheme S 2. Synthesis of Cu@BTTAA-NPs (7). Reagents and conditions: (a) BTTAA (15 eqiv.), Oxyme (15 equiv.), DIC (15 equiv.), DMF (2h, 60ºC, 1200 rpm); (b) CuBr (50mM), DMF (o/n, RT, 1200 rpm).

Naked-NPs (NK-NPs) were first conditioned by washing them three times with 1 mL of DMF each time through suspension-centrifugation cycles (13,400 rpm, 3 min). Next, the copper ion chelator was conjugated, BTTAA ligand **5** (15 eq) was dissolved in DMF (1 mL) with oxyma (15 eq) and DIC (15 eq). The mixture was stirred at RT for 10 min. Then, the solution was added to dry NK-NPs, and the suspension was left to stir at 1,400 rpm at 60 °C for 2 h. Subsequently, the NPs in suspension were washed with three successive suspension-centrifugation cycles (13,400 rpm, 3 min) to obtain **BTTAA-NPs** (**6**). Next, 1 mL of CuBr (50mM) solution in DMF was added to dry **BTTAA-NPs (6)**, which were then suspended. The suspension was stirred at 1,000 rpm at RT for 14 h in the dark. Afterwards, the NPs were washed with three cycles of suspension-centrifugation with DMF (13,400 rpm, 3 min), then other three cycles in MeOH, and finally resuspended in mQ H_2O to obtain **Cu@BTTAA-NPs (7)**.

7.2. Synthesis of Cu@Cy5-BTTAA-NPs (11)

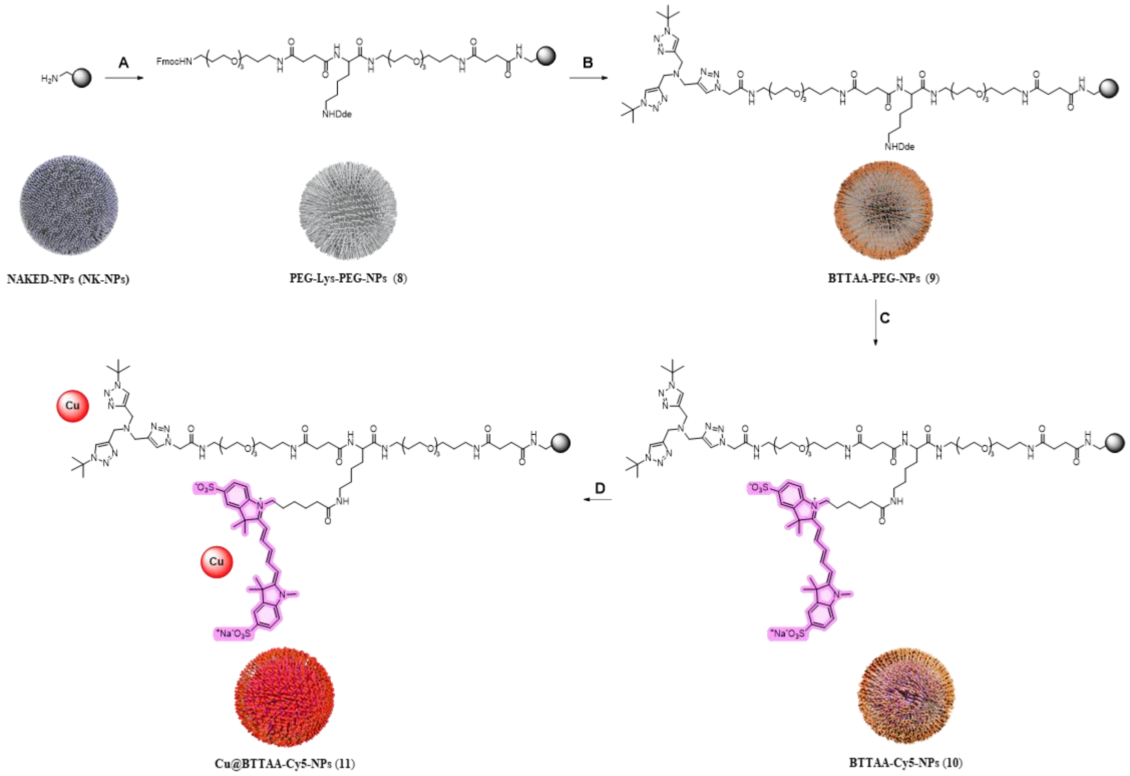

Scheme S 3. **Synthesis of Cu@BTTAA-Cy5-NPs (11). Reagents and conditions: A**: (i) Fmoc-PEG-OH (15 eq), Oxyma (15 eq), DIC (15 eq), DMF, 2 h, 60 °C; (ii) Fmoc Deprotection: 20% piperidine/DMF, 3 x 20 min; (iii) Fmoc-LysDde(OH) (15 eq), Oxyma (15 eq), DIC (15 eq), DMF, 2 h, 60 °C; **B**: (ii) Fmoc Deprotection: 20% piperidine/DMF, 3 x 20 min; (iv) BTTAA (15 eqiv.), Oxyme (15 equiv.), DIC (15 equiv.), DMF (2h, 60ºC, 1200 rpm); **C**: (v) Dde Deprotection: Hydroxylamine.HCl, Imidazole, NMP, 2 x 1 h, 25 ºC; (vi) Sulfo-Cy5-NHS (1 eq), DIPEA (1 eq), DMF, 15 h, 25 ºC, ; **D**: (vii) CuBr (50mM), DMF (o/n, RT, 1200 rpm).

Naked-NPs were washed in DMF (1 mL x 3 times) and suspended in DMF (1 mL). Separately, the Fmoc-PEG spacer (15 eq.) was dissolved in DMF (1 mL), then oxyma (15 eq.) was added and the solution mixture mixed for 4 minutes at r.t. before the addition of DIC (15 eq.) and mixed for 8-10 minutes at 25ºC. The solution mixture was then added to Naked NPs and suspension mixed on the Thermomixer at 1400 rpm for 2 hours at 60°C. Fmoc deprotection was achieved by treating NPs with 20% piperidine/DMF. This PEG functionalised NPs (1 mL; 1 eq.) were washed in DMF (1 mL x 3 times) and suspended in DMF (1 mL). Separately, Fmoc-Lys(Dde)OH (15 eq.) was dissolved in DMF (1 mL), then oxyma (15 eq.) was added and the solution mixture mixed for 4 minutes at r.t. before the addition of DIC (15 eq.) and mixed for 8-10 minutes at r.t. The solution mixture was then added to this NH_2-PEG-NPs and suspension mixed on the Thermomixer at 1400 rpm for 2 hours at 60°C. Then this step was repeated to Fmoc deprotection and to introduce one unit PEG spacer to obtain **Fmoc-Dde-NPs (8)**. Then, Fmoc deprotection protocol and next, the copper ion chelator was conjugated, BTTAA ligand **5** (15 eq) was dissolved in DMF (1 mL) with oxyma (15 eq) and DIC (15 eq). The mixture was stirred at RT for 10 min. Then, the solution was added to NH2-Dde-NPs, and the suspension was left to stir at 1,400 rpm at 60 °C for 2 h. Subsequently, the NPs in suspension were washed with three successive suspension-centrifugation cycles (13,400 rpm, 3 min) to obtain **BTTAA-Dde-NPs (9)**. Then, Dde deprotection was facilitated by treating **BTTAA-Dde-NPs (9)** with the Dde deprotection solution mixture (1.25 g (1.80 mmol) of NH2OH.HCl and 0.918 g (1.35 mmol) of imidazole were

suspended in 5 mL of NMP, and the mixture was sonicated until complete dissolution). Just before reaction, 5 volumes of this solution were diluted with 1 volume of DMF (1 mL) for 1 hour at r.t. on a rotary wheel, then NPs were washed with DMF (1 mL). NPs were obtained by centrifugation and subsequently washed with DMF (3 x 1 mL), methanol (3 x 1 mL), deionised water (3 x 1 mL) and finally DMF (3 x 1 mL). Next, 50 µL of a sulfo-Cy5-NHS ester solution (1 eq) was mixed separately with DIPEA (1 eq) before being added to dry BTTAA-NH2-NPs, which were then suspended. The suspension was stirred at 1,000 rpm at RT for 14 h in the dark. Afterwards, the NPs were washed with three cycles of suspension-centrifugation with DMF (13,400 rpm, 3 min), then other three cycles in MeOH, and finally resuspended in mQ H_2O to obtain **BTTAA-Cy5-NPs (10)**. Then, as we described previously for the synthesis of **Cu@BTTAA-NP (7)**, 1 mL of CuBr (50mM) solution in DMF was added to dry **BTTAA-Cy5-NPs (10)**, which were then suspended. The suspension was stirred at 1,000 rpm at RT for 14 h in the dark. Afterwards, the NPs were washed with three cycles of suspension-centrifugation with DMF (13,400 rpm, 3 min), then other three cycles in MeOH, and finally resuspended in mQ H_2O to obtain **Cu@BTTAA-Cy5-NPs (11)**.

7.3. Characterization of Cu@NPs

7.3.1. DLS and ZETA POTENCIAL

The average particle size, size distribution, and zeta potential of **Cu@BTTAA-Cy5-NPs (11)** were analyzed using dynamic light scattering (DLS). These measurements were performed with a Benano 90 Zeta, using biological grade water as the medium. A disposable cuvette was used for size determination, while a transparent disposable cuvette was used for zeta potential measurements. A sample of 1 µL of the nanoparticle suspension is taken and suspended in 999 µL of deionized water and finally placed in a disposable cuvette to measure the hydrodynamic diameter by DLS or ζ-potential.

7.3.2. TEM Analysis

Preparation of samples for TEM-analysis: for the TEM-analysis, 10 µL of a nanoparticle suspension (in ethanol) are added to 0.5 mL ethanol absolute and suspended via sonication. The TEM analysis was performed to confirm the presence of Cu on the **Cu@BTTAA-NPs** (**7**) and **Cu@BTTAA-Cy5-NPs** (**11**).

7.3.3. XPS

To prepare a thin layer of dried nanoparticles (NPs), 1 mL of NPs were washed in ethanol and applied dropwise to a coverslip and allowed to dry air. XPS spectra were recorded using a Kratos Axis Ultra-DLD X-ray photoelectron spectrometer equipped with a monochromatic Al X-ray source. Analyses were performed on nanoparticle powder samples. General spectra were collected using a pass energy of 160 eV with the X-ray source operating at 75 W. High resolution spectra were obtained using a pass energy of 20 eV with the X-ray source operating at 225 W.

7.3.4. ICP-MS

Inductively coupled plasma mass spectrometry (ICP-MS) is a robust analytical technique used for elemental and isotopic analysis. It operates by atomizing and ionizing the sample within a high-energy argon plasma.[4] Cu@NPs **7** and **11** was measured by inductively coupled plasma mass spectrometry (ICP-MS). 1 mL of **Cu@BTTAA-NPs (7)** was collected for ICP-MS analysis of copper content using a Perkinelmer NexION 300D

ICP-MS. The measured c/s (count/second) was converted to the copper concentration using the standard calibration curve. Concentration of copper was 2580.138 ppb in **Cu@BTTAA-NPs (7)**. 1 mL of **Cu@BTTAA-Cy5-NPs (11)** was collected for ICP-MS analysis of copper content using a Perkinelmer NexION 300D ICP-MS. The measured c/s (count/second) was converted to the copper concentration using the standard calibration curve. Concentration of copper was 20 ppm in **Cu@BTTAA-Cy5-NPs (11)**.

7.3.5. EDX-HRTEM

Sample preparation for HRTEM and EDX analysis: a 5 µL sample of NPs was suspended in absolute ethanol (1 mL), treated with copper and deposited on a charcoal-lined grid.

7.3.6. Estimation of copper content in each NP

Using the data obtained from ICP-MS to determine the amount of copper in our batch of nanoparticles, along with the previously obtained value for nanoparticle concentration (NPs/µL) from the spectrophotometric method, we can estimate the copper content per nanoparticle by applying the following expression:

$$mmol\ Cu = \frac{C_{Cu} \cdot N_{NP}}{1000 \cdot M_{Cu} \cdot N_{total}}$$

where:

C_{Cu}: the copper concentration in ppm,
N_{NP}: Specific number of NP we're considering
M_{Cu}: the molar mass of copper,
N_{total}: the total number of NP in 1 mL

7.3.7. Detarmination of fluorophore concentration

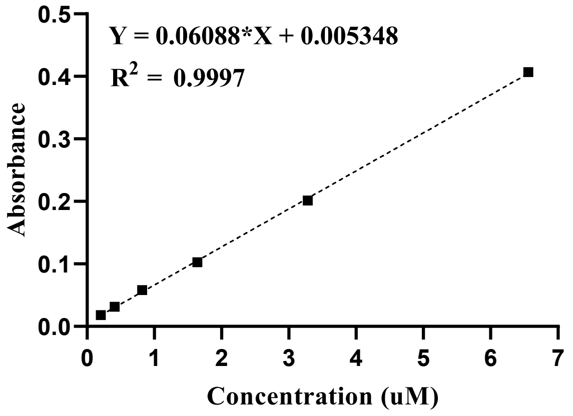

Figure S16. Calibration standard curve of Cy5 by spectrophotometry.

The loading capacity (LC) of Cy5 was then calculated using the following formula:

$$Loading\ Capacity\ (LC) = \frac{C_{Cy5} \cdot conjugated\ on\ NP\ surface}{\text{Loading of free amine groups@on nanoparticle surface}}$$

8. Synthesis of Prodrug substrate 5-azidobenzene-1,3-diol (13)[5]

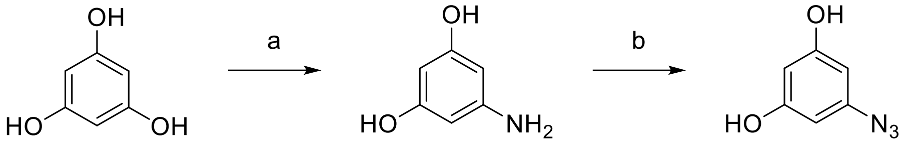

12 **13**

Scheme S 4. Synthesis of BTTAA ligand. Reagent and conditions: a) $NH_3.H_2O$, argon atmosphere, room temperature, 24 hours; b) HClconc:H_2O (1:1), $NaNO_2$, NaN_3, 0 ºC, 40 min.

Synthesis of 5-Aminoresorcinol hydrochloride (12)

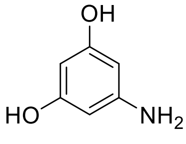

12

Phloroglucinol (10.0 g, 79.5 mmol) and conc. $NH_3{\cdot}H_2O$ (78 mL) was mixture under Argon atmosphere. The obtained solution was stirred at room temperature for one day, and then the solvent was distilled off under reduced pressure. To the obtained solution, 6 N HCl was added under ice cooling to form a hydrochloric acid salt, and the solvent was distilled off under reduced pressure, and then the resultant was purified with MeOH/DCM reprecipitation, to give the targeted compound as a yellow-green solid. **^{1}H NMR** (400 MHz, MeOD) δ 6.32 (s, 1H), 6.29 (s, 2H). **^{13}C NMR (101 MHz, MeOD)** δ 161.08, 133.00, 103.85, 102.38. **HRMS Calculated for [M+H]$^+$**: 126.0550, **Found:** 126.0526

Synthesis of 5-azidobenzene-1,3-diol (13)

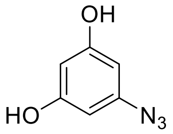

13

5-Aminoresorcinol hydrochloride (1.105 g, 6.840 mmol, 1.000 eq) was added in 2.5 mL distilled water and 2.5 mL conc. HCl under 0 °C. $NaNO_2$ (0.472 g, 6.840 mmol, 1.000 eq) in 2.5 mL H_2O was added to the solution slowly (> 5 min). 10 min later, NaN_3 (0.489 g, 7.524 mmol, 1.100 eq) in 2.5 mL H_2O was added to the solution and the solution was stirring for 40 min under 0 °C. The solution extracted with EtOAc (3 x 20 mL). The combined organic layers were washed with brine (1 x 20 mL), dried over anhydrous MgSO4, concentrated in vacuo and the crude product was chromatographed with Hexane/EtOAc as eluant, to afford the light-yellow crystal. **^{1}H NMR** (400 MHz, DMSO-d6) δ 9.55 (s, 2H), 6.02 (s, 1H), 5.92 (s, 2H). **^{13}C NMR (101 MHz, DMSO-d6)** δ 159.51, 140.69, 99.76, 97.19. **HRMS Calculated for [M+H]$^+$**: 150.0309, **Found:** 150.0306.

9. General procedure for the copper-NP catalyzed 1,3-dipolar cycloaddition

In an eppendorf loaded with Copper-NP, previously centrifugate and disperse in 100 uL of methanol, a mixture of alkyne (0.01 mmol, 1.000 eq) and azide (0.01 mmol, 1.000 eq) were added. Then 100 uL of a solution of sodium ascorbate 0.01M (0.001 mmol, 0.1 eq) in water was added to the mixture. The heterogeneous mixture was stirred in thermoshaker at room temperature. Monitoring the reaction by TLC. When the reaction is finished, the mixture is centrifugated during 5 minutes at 14000 rpm, and the NPs were washed three times with MeOH. The solvent was transferred to a 10 mL round flask, and the mixture was evaporated and purified by a short flash chromatography to get the corresponding click product.

10. General procedure for recyling copper-NP catalyzed 1,3-dipolar cycloaddition.

The **Cu@BTTAA-Cy5-NPs (11**) used in each cycle, were previously whased three times with MeOH, and then were subjected to the general procedure described above. In an eppendorf loaded with Copper-NP, previously centrifugate and disperse in 100 uL of methanol, a mixture of alkyne (0.01 mmol, 1.000 eq) and azide (0.01 mmol, 1.000 eq) was added. Then 100 uL of a solution of sodium ascorbate 0.01M (0.001 mmol, 0.1 eq) in water was added to the mixture. The heterogeneous mixture was stirred in thermoshaker at room temperature. Monitoring the reaction by TLC. When the reaction is finished, the mixture is centrifugated during 5 minutes at 14000 rpm, and the NPs were washed three times with MeOH. The solvent was transferred to a 10 mL round flask, and the mixture was evaporated and purified by a short flash chromatography to get the corresponding click product.

11. Determination of Copper by UV-Vis spectroscopy analysis.

The procedure is an adapted version of the method reported by A. J. Brenner and E. D. Harris.[6–8]

Calibration curve

The calibration curve was prepared by serially diluting a 100 µg/mL anhydrous $CuSO_4$ using a 90:10 (v/v) mixture of ultrapure water and methanol to mimic the sample matrix from 100 ppm to 1 ppm. For each calibration point, 25 µL of the standard solution was combined with 200 µL of Reagent A (from a commercially available Pierce™ BCA Protein Assay Kit) and 25 µL of 10 mM sodium ascorbate. The reaction mixtures were incubated at 37 °C for 30 minutes or at room temperature for 2 hours minutes to allow complete reduction of Cu(II) to Cu(I) and formation of the Cu(I)-BCA complex. Absorbance was then measured at 562 nm using a NanoQuant UV-Vis spectrophotometer and blank values (0 µg/mL) were subtracted. The resulting data were plotted against Cu^{2+} concentrations, and a linear regression model was fitted to generate the calibration curve, which was subsequently employed for the quantification of copper in experimental samples.

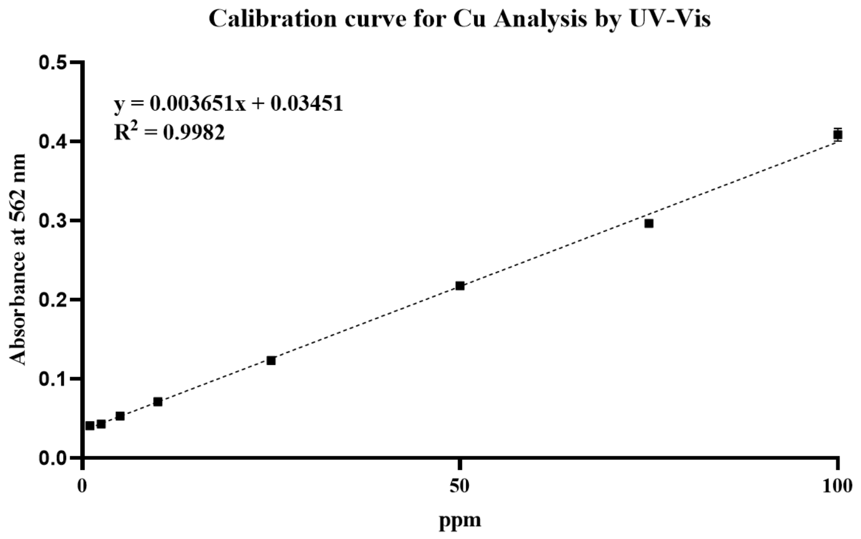

Figure S17. Calibration curve for copper analysis by UV-Vis.

Table S7. Absorbance Measurements and Determination of Cu^{2+} in supernatants and compound 14c in recyclability experiment.

Method Performance	
**LoD (ppm)**	1.778
**LoQ (ppm)**	5.387

Sample	Absorbance	Interpolated Concentration (ppm)	Copper Detected	Copper Quantified
**Blank**	0.037	0.064	No	No
**SN-1**	0.003	-0.860	No	No
**14c_Cycle 1**	0.046	0.319	No	No
**SN-2**	0.007	-0.748	No	No
**14c_Cycle 2**	0.084	1.362	No	No
**SN-3**	0.015	-0.526	No	No
**14c_Cycle 3**	0.071	1.001	No	No
**SN-4**	0.008	-0.730	No	No
**14c_Cycle 4**	0.063	0.770	No	No
**SN-5**	0.008	-0.728	No	No
**14c_Cycle 5**	0.077	1.168	No	No
**SN-6**	0.002	-0.881	No	No
**14c_Cycle 6**	0.116	2.222	Yes	No
**SN-7**	0.040	0.160	No	No
**14c_Cycle 7**	0.134	2.737	Yes	No

SN: supernantant from nanoparticles washing after each recyclability cycle**. 14c:** Compound after each recyclability cycle**.** 1-7 cycles of recyclability.

12. Leaching test using hot-filtration.[9]

A model reaction between 4-azidoanisole and phenylacetylene was carried out under optimized conditions and interrupted after 5 minutes. The reaction mixture was

centrifuged to remove the catalyst, and the resulting supernatant was stirred under identical conditions for an additional 60 minutes. No significant further product formation was observed by TLC analysis. After purification, the isolated yield of the triazole was 45%, corresponding to the conversion achieved prior to catalyst removal. These findings confirm that the reaction does not proceed in the absence of the solid catalyst, strongly supporting a truly heterogeneous mechanism with no appreciable copper leaching into solution.

Table S8. Modified hot filtration test indicating absence of catalyst leaching.

Reaction Condition	Reaction Time	Conversion / Yield
Phase 1: With Catalyst	5 minutes	45%
Phase 2: Without Catalyst	60 minutes	45%

13. Relationship between volumen of Cu@BTTAA-NPs (7) and Cu@BTTAA-Cy5-NPs (11) and the concentration of copper.

Based on the copper concentration measured by ICP-MS for each type of nanoparticle, we can extrapolate and estimate the copper concentration according to the volume of nanoparticles used, as shown in the **Table S8**.

Table S9. Relationship between volumen of NPs and the concentration of copper.

Volumen of Cu@BTTAA-NPs (7) (uL)	Volumen of Cu@BTTAA-Cy5-NPs (11) (uL)	[ppm Cu]
38.76	5	0.1
193.80	25	0.5
387.60	50	1
581.40	75	1.5
775.19	100	2

14. Characterization of product scope Table 3

1,4-diphenyl-1H-1,2,3-triazole (14a). ^{1}**H NMR** (400 MHz, $CDCl_3$) δ 8.20 (s, 1H), 7.92 (d, *J* = 8.4 Hz, 2H), 7.80 (d, *J* = 8.5 Hz, 2H), 7.55 (t, *J* = 7.8 Hz, 2H), 7.50 – 7.43 (m, 3H), 7.37 (t, *J* = 6.8 Hz, 1H). ^{13}**C NMR** (101 MHz, $CDCl_3$) δ 148.57, 137.24, 130.41, 129.93, 129.07, 128.92, 128.58, 126.01, 120.69, 117.72. **HRMS Calculated for [M+Na]$^+$**: 244.0845, **Found:** 244.0856.

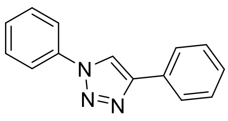

14a

4-(4-phenyl-1H-1,2,3-triazol-1-yl)aniline (14b). ^{1}**H NMR (**400 MHz, $CDCl_3$**)** δ 8.06 (s, 1H), 7.90 (d, J = 8.3 Hz, 2H), 7.52 (d, J = 8.8 Hz, 2H), 7.45 (t, J = 7.5 Hz, 2H), 7.35 (t, J = 7.4 Hz, 1H), 6.78 (d, J = 8.8 Hz, 2H), 3.90 (br., 2H)**.** ^{13}**C NMR** (101 MHz, $CDCl_3$) δ 148.13, 147.27, 130.69, 129.01, 128.82, 128.36, 125.93, 122.43, 117.94, 115.44. **HRMS Calculated for [M+H]$^+$**: 237.1135, **Found:** 237.1141.

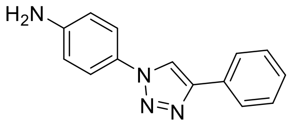

14b

1-(4-methoxyphenyl)-4-phenyl-1H-1,2,3-triazole (14c). ^{1}**H NMR** (400 MHz, $CDCl_3$) δ 8.11 (s, 1H), 7.91 (d, J = 8.1 Hz, 2H), 7.69 (d, J = 9.1 Hz, 2H), 7.46 (t, J = 7.8 Hz, 2H), 7.36 (t, J = 7.4 Hz, 1H), 7.04 (d, J = 9.1 Hz, 2H), 3.88 (s, 3H). ^{13}**C NMR** (101 MHz, $CDCl_3$) δ 160.01, 148.37, 130.71, 130.54, 129.04, 128.47, 125.97, 122.34, 117.97, 114.95, 55.79**. HRMS Calculated for [M+H]$^+$**: 252.1131, **Found:** 252.1149.

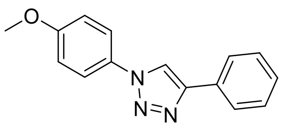

14c

4-phenyl-1-(4-(trifluoromethyl)phenyl)-1H-1,2,3-triazole (14d). ^{1}**H NMR** (500 MHz, $CDCl_3$) δ 8.25 (s, 1H), 7.97 (d, J = 8.5 Hz, 2H), 7.92 (d, J = 7.5 Hz, 2H), 7.84 (d, J = 8.9 Hz, 2H), 7.49 (t, J = 7.6 Hz, 2H), 7.40 (t, J = 7.4 Hz, 1H). ^{13}**C NMR** (126 MHz, $CDCl_3$) 149.12, 139.45, 130.66, 129.34, 128.90, 128.02, 127.14, 125.82, 122.75 120.55**. HRMS Calculated for [M+H]$^+$**: 290.0900, **Found:** 290.0907.

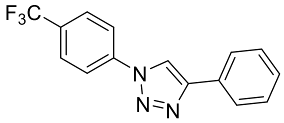

14d

(1-phenyl-1H-1,2,3-triazol-4-yl)methanol (14e). ^{1}**H NMR** (400 MHz, $CDCl_3$) δ 7.99 (s, 1H), 7.71 (d, J = 8.3 Hz, 2H), 7.52 (t, J = 7.6 Hz, 2H), 7.44 (t, J = 7.4 Hz, 1H), 4.89 (s, 2H). ^{13}**C NMR** (101 MHz, $CDCl_3$) δ 148.48, 137.13, 129.92, 129.00, 120.75, 120.20, 56.64. **HRMS Calculated for [M+H]$^+$**: 176.0818, **Found:** 176.0821.

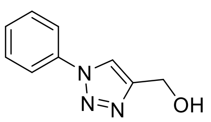

14e

(1-(4-methoxyphenyl)-1H-1,2,3-triazol-4-yl)methanol (14f). ^{1}**H NMR** (500 MHz, CDCl3) δ 7.89 (s, 1H), 7.62 (d, J = 9.0 Hz, 2H), 7.03 (d, J = 9.2 Hz, 2H), 4.89 (s, 2H), 3.87 (s, 3H), 3.65 (s, 1H). ^{13}C NMR (126 MHz, CDCl3) δ 160.08, 147.80, 130.73, 122.31, 120.12, 114.88, 56.83, 55.70**. HRMS Calculated for [M+H]$^+$**: 206.0924, **Found:** 206.0916.

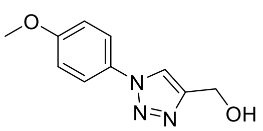

14f

(1-(4-aminophenyl)-1H-1,2,3-triazol-4-yl)methanol (14g). ^{1}**H NMR** (500 MHz, MeOD) δ 8.24 (s, 1H), 7.48 (d, J = 8.9 Hz, 2H), 6.82 (d, J = 8.7 Hz, 2H), 4.74 (s, 2H), 3.66 (s, s,

3H, NH_2 and OH). **^{13}C NMR** (126 MHz, MeOD) δ 149.12, 147.92, 127.19, 121.65, 120.74, 69.46, 49.95. **HRMS Calculated for [M+H]$^+$**: 191.0927, **Found:** 191.0930.

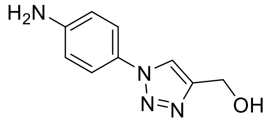

14g

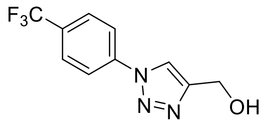

14h

(1-(4-(trifluoromethyl)phenyl)-1H-1,2,3-triazol-4-yl)methanol (14h). ^{1}H NMR (500 MHz, MeOD) δ 8.59 (s, 1H), 8.12 (d, J = 8.4 Hz, 2H), 7.92 (d, J = 8.4 Hz, 2H), 4.79 (s, 2H), 3.65 (s, 1H). **^{13}C NMR** (126 MHz, MeOD) δ 148.45, 138.92, 129.51, 126.16, 120.44, 119.82, 119.71, 69.52**. HRMS Calculated for [M+H]$^+$**: 244.0692, **Found:** 244.0695.

(1-phenyl-1H-1,2,3-triazol-4-yl)methanamine (14i). ^{1}H NMR (400 MHz, DMSO-d6) δ 8.78 (s, 1H), 7.88 (d, J = 9.0 Hz, 2H), 7.66 – 7.44 (m, 5H), 4.08 (s, 2H)**. HRMS Calculated for [M+H]$^+$**: 175.0978, **Found:** 175.0978.

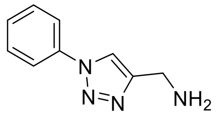

14i

1-phenyl-1H-1,2,3-triazole-4-carboxylic acid (14j). ^{1}H NMR (500 MHz, DMSO-d6) δ 13.63 (s, 1H), 9.53 (s, 1H), 8.13 (d, J = 7.5 Hz, 2H), 7.77 (t, J = 8.2 Hz, 2H), 7.69 (t, J = 7.4 Hz, 1H). **^{13}C NMR** (126 MHz, DMSO-d6) δ 162.00, 141.13, 136.65, 130.35, 129.66, 127.49, 120.99. **HRMS Calculated for [M+H]$^+$**: 190.0611, **Found:** 190.0565

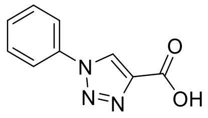

14j

7-hydroxy-3-(4-phenyl-1H-1,2,3-triazol-1-yl)-2H-chromen-2-one (14k). 1H NMR (500 MHz, DMSO-d6) δ 10.93 (s, 1H), 8.99 (s, 1H), 8.65 (s, 1H), 7.95 (d, J = 7.6 Hz, 2H), 7.76 (d, J = 8.5 Hz, 1H), 7.48 (t, J = 7.5 Hz, 2H), 7.37 (t, J = 7.4 Hz, 1H), 6.92 (dd, J = 6.8, 4.3 Hz, 1H), 6.87 (s, 1H). **13C NMR** (126 MHz, DMSO-d6) δ 162.57, 156.36, 154.77, 146.47, 136.82, 131.06, 130.08, 129.04, 128.27, 125.41, 122.19, 119.25, 114.35, 110.35, 102.23. **HRMS Calculated for [M+H]$^+$**: 306.0879, **Found:** 306.0874.

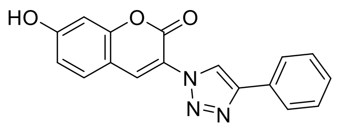

14k

5-(4-phenyl-1H-1,2,3-triazol-1-yl)benzene-1,3-diol (14l). ^{1}H NMR (400 MHz, DMSO-d6) δ 9.86 (s, 2H), 9.18 (s, 1H), 7.95 (d, *J* = 7.4 Hz, 2H), 7.49 (t, *J* = 7.8 Hz, 2H), 7.38 (t, *J* = 7.4 Hz, 1H), 6.81 (d, *J* = 2.1 Hz, 2H), 6.35 (t, *J* = 2.1 Hz, 1H). **^{13}C NMR** (101 MHz, DMSO-d6) δ 159.33, 147.03, 138.06, 130.31, 128.94, 128.15, 125.32, 119.43, 102.46, 98.31. **HRMS Calculated for [M+H]$^+$**: 254.0924, **Found:** 254.0920.

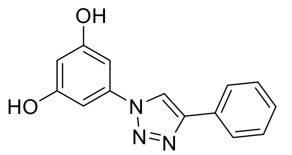

14l

15. NMR of compound 14l

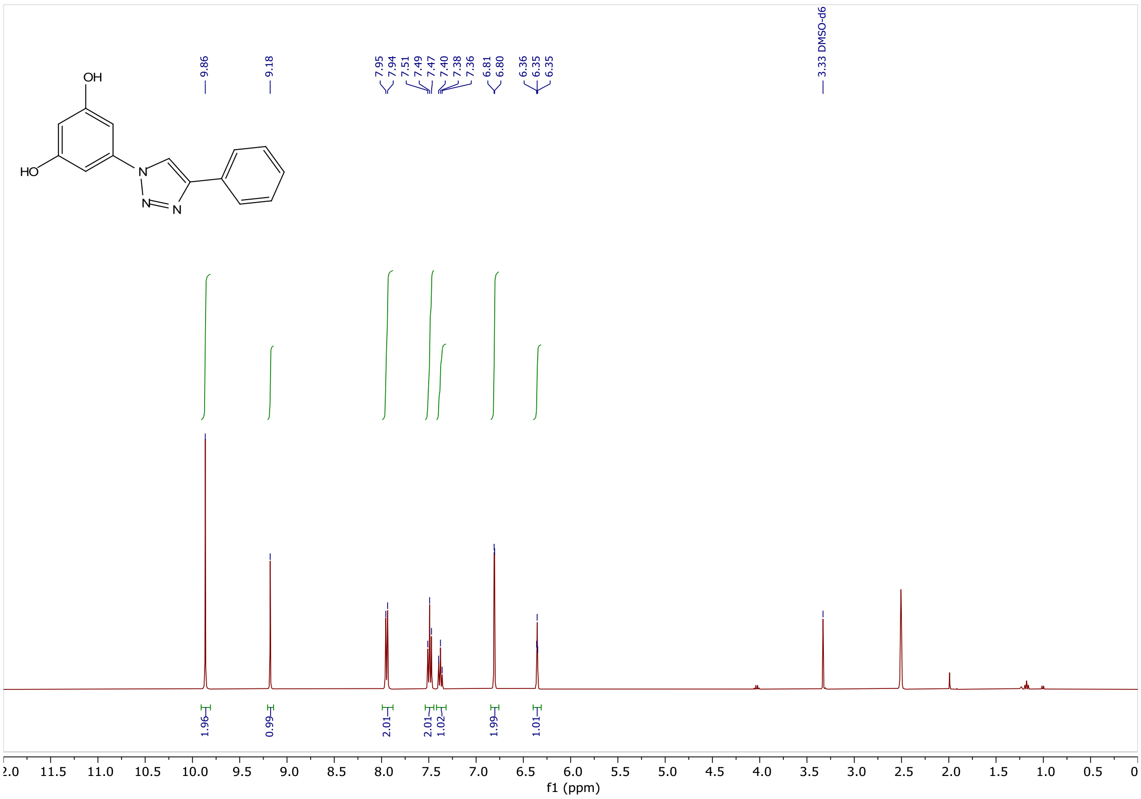

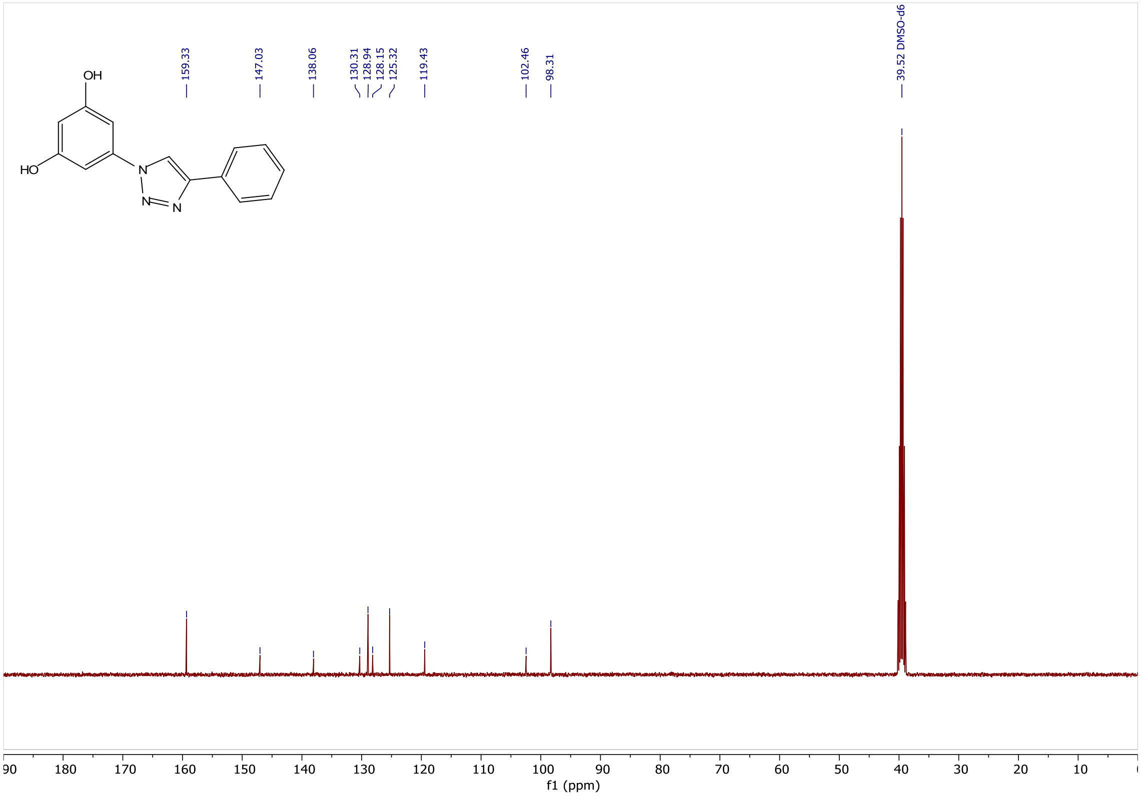

16. General protocols for evaluation of intracellular activity and safety of nanocatalyst

16.1. Relationship between number of Cu@BTTAA-Cy5-NPs (11) and the amount of copper.

Taking into account the calculation carried out to estimate the amount of copper in each nanoparticle, calculated in the previus section, the amount of copper added to the reaction can be easily extrapolated knowing the exact number of nanoparticles that are added (**Table S10**).

Table S10. Relationship between the number of nanoparticles and the amount of copper

Number of Cu@BTTAA-Cy5-NPs (11)	fmol Cu
1	0.000463
1000	0.463
5000	2.32
10000	4.63
15000	6.95
20000	9.27
25000	11.6
30000	13.9
40000	18.5
50000	23.2
75000	34.8
100000	46.3
125000	57.9
150000	69.5
300000	139
500000	232
1000000	463

16.2. Cellular uptake by flow cytometry

After incubating the cells with different amounts of **Cu@BTTAA-Cy5-NPs (11)**, the medium was removed and the cells were rinsed with 1x PBS before detachment with trypsin-EDTA at 37°C for 5 minutes. Each sample was then fixed in 2% paraformaldehyde (PFA) for 10 minutes at room temperature in the dark. Samples were then analysed using a FACSCanto II flow cytometer. Each experiment was performed in triplicate for different incubation ratios and times and was repeated at least three times.

The study of the uptake of **Cu@BTTAA-Cy5-NPs (11)** were carried out using the following amount of copper per cell contained in NPs: 0.02, 0.04, 0.11, 0.23, 0.23, 0.34, 0.46, 1.16, 2.32, 3.48, 4.63 and 69.5 fmol. Dot plots and cytometry statistics were generated using FlowJo software. Graphs and statistical analysis were performed using GraphPad software as described below. The percentage of cells containing NPs was plotted against the cell/NP ratio using a bar graph display to determine statistically significant differences. These differences were analysed using ordinary one-way ANOVA, specifically Dunnett's multiple comparison test, to compare the different levels of nanofection based on the number of internalised nanoparticles.

16.3. Cellular uptake by confocal microscopy

MDA-MB-231 cells were rinsed with 1x PBS, detached using trypsin/EDTA, counted and diluted in the appropriate medium to a final concentration of 10^5 cells per mL. A total of 500 µL of each cell suspension was seeded onto poly-L-lysine-precoated glass coverslips in 24-well plates and incubated for 15 hours. The medium was then replaced with fresh medium containing 2.32 fmol of copper in **Cu@BTTAA-Cy5-NPs (11)**, **BTTAA-Cy5-NPs (10**), and 2.32 fmol of compound **14L**. After the specified incubation time, the medium was removed, and the cells were washed with 1X PBS before being fixed in 4% paraformaldehyde for 30 minutes at room temperature. The fixed cells were then rinsed with 1X PBS and mounted using ProLong Gold mounting medium (Life Technologies). Images were captured using a ZEISS LSM 710 confocal laser microscope equipped with a DIC Plan-Apochromat 63x oil immersion objective (1.40 numerical aperture) and ZEN 2010 software. Image analysis was performed using ZEN 2012 Blue Edition or ImageJ version 1.49b (open source software). Fluorescent NPs and cells were visualised using excitation from a 633 nm HeNe laser line (5.0 mW) at 7% power for NPs and a 405 nm diode laser line (30.0 mW) at 2.8% power to detect fluorescent cells due to the formation of the 14K compound, with an Airy unit (AU) of 1.00. Each experiment was performed in duplicate and repeated three times.

16.4. Cell experiment quantification through LC/MS analysis

Calibration curve of compound 14k

Stock solutions of compound 14k at various concentrations (0.094–1.5 µM) were prepared in methanol. To generate the calibration curve, the peak area ratios of compound 14k obtained from HPLC-UV spectra at 254 nm were plotted against their corresponding concentrations.

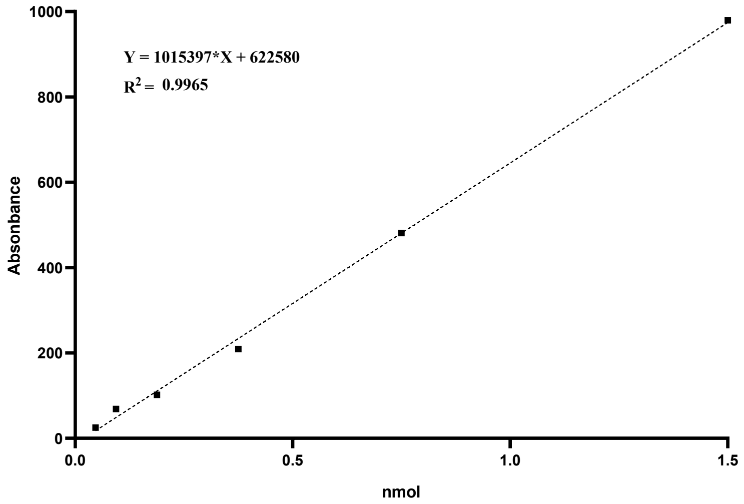

Figure S18. Calibration curve of compound **14k**

Quantification of compound 14k formed via intracellular CuAAC reaction catalysed by Cu@BTTAA-Cy5-NP (11)

MDA-MB-231 cells are seeded at a density of 1 × 10^6 cells per T25 flask and incubated for 18 hours at 37 °C. Following incubation, nanoparticles (NPs) are added at a ratio of 50,000 NPs per cell in 6 mL of culture medium. Cells are then incubated for an additional 3 hours.

After NP treatment, the medium is removed, and cells are washed twice with PBS. Subsequently, compounds **A-5**, **Phe** and sodium ascorbate are added in 3 mL of solution, and cells are incubated for a further 3 hours. After incubation, each well is washed twice with PBS.

To extract intracellular content, 4 mL of cold methanol are added to each well, and the plates are incubated at −20 °C for 10 minutes. Cells are then scraped thoroughly, and the entire content from each well is transferred to Eppendorf tubes. Samples are centrifuged at 15,000 rpm for 10 minutes at 4 °C. The resulting supernatant is collected, dried in a desiccator and analysed.

For the analysis, the final samples were dissolved in 1 mL of methanol and injected in a Waters Acquity UPLC I-Class.

Table S11. Quantification of compound **14k** formed via intracellular CuAAC reaction catalysed by Cu@BTTAA-Cy5-NP (11)

Sample	Absorbance (Area)	nmol
Compound 14k	342.787	0.542

Calibration curve of compound 14l

Stock solutions of compound 14l at various concentrations (0.094–1.5 µM) were prepared in methanol. To generate the calibration curve, the peak area ratios of compound 14l obtained from HPLC-UV spectra at 254 nm were plotted against their corresponding concentrations.

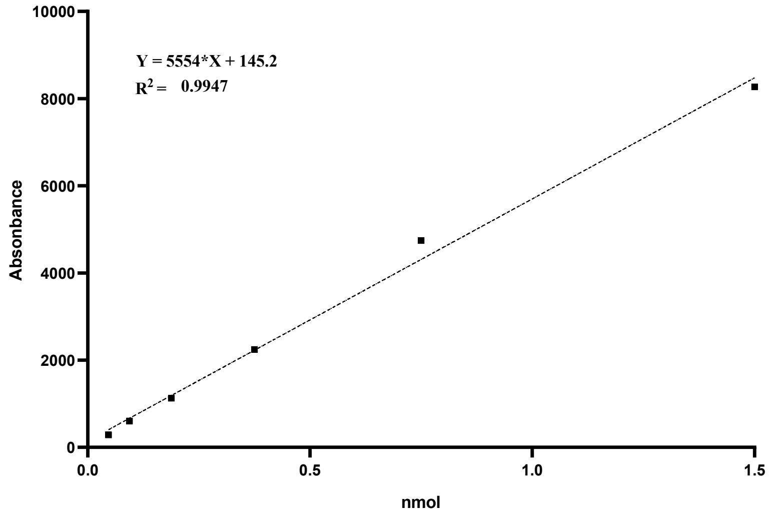

Figure S19. Calibration curve of compound **14l**

Quantification of compound 14l formed via intracellular CuAAC reaction catalysed by Cu@BTTAA-Cy5-NP (11)

MDA-MB-231 cells are seeded at a density of 1 × 10^6 cells per T25 flask and incubated for 18 hours at 37 °C. Following incubation, nanoparticles (NPs) are added at a ratio of 50,000 NPs per cell in 6 mL of culture medium. Cells are then incubated for an additional 3 hours.

After NP treatment, the medium is removed, and cells are washed twice with PBS. Subsequently, compounds **13**, **Phe** and sodium ascorbate are added in 3 mL of solution, and cells are incubated for a further 3 hours. After incubation, each well is washed twice with PBS.

To extract intracellular content, 4 mL of cold methanol are added to each well, and the plates are incubated at −20 °C for 10 minutes. Cells are then scraped thoroughly, and the entire content from each well is transferred to Eppendorf tubes. Samples are centrifuged at 15,000 rpm for 10 minutes at 4 °C. The resulting supernatant is collected, dried in a desiccator and analysed.

For the analysis, the final samples were dissolved in 1 mL of methanol and injected in a Waters Acquity UPLC I-Class.

Table S12. Quantification of compound **14l** formed via intracellular CuAAC reaction catalysed by Cu@BTTAA-Cy5-NP (**11**)

Sample	Absorbance (Area)	nmol
Compound **14l**	2788.082	0.476

16.5. Cell viability

The cytotoxic effects of **NK-NP**, **Cu@BTTAA-NPs (7)** and **Cu@BTTAA-Cy5-NPs (11)**, click reagents and compounds **14k** and **14l** were assessed using the resazurin assay protocol. Cells were plated in 96-well plates at a density of 2.5 × 10^3 MDA-MB-231 cells per well and incubated for 15 hours. The medium was then replaced with fresh medium containing the test samples at varying concentrations. After 96 hours of incubation, the medium was removed and replaced with fresh phenol red-free complete medium. Fluorescence readings were performed according to the manufacturer's instructions, with fluorescence intensity correlating with the number of viable cells. Cell viability was expressed as a percentage relative to untreated control cells (set at 100%). Each plate included control wells to measure the fluorescence of the culture medium containing NPs and different reagents in the absence of cell

16.6. Safety assessment of nanoparticles

16.6.1. Nanoparticle bacterial contamination assessment

The assessment was carried out using agar Petri dishes. **NK-NP**, **Cu@BTTAA-NPs (7)** and **Cu@BTTAA-Cy5-NPs (11)** was applied to the dishes using an inoculation loop, followed by incubation at 37 °C for three days. Dishes were checked daily for bacterial colony growth. As controls, one Petri dish was treated with PBS as a negative control, while another was inoculated with 10 µL of Escherichia coli (300 CFU) as a positive control.

16.6.2. Endotoxin test

The endotoxin assay was performed using the Pierce LAL Chromogenic Endotoxin Quantitation Kit. **NK-NP**, **Cu@BTTAA-NPs (7)** and **Cu@BTTAA-Cy5-NPs** (**11**) were incubated with the LAL reagent at 37 °C for 10 minutes. After incubation, the chromogenic substrate solution was added and incubated for a further 6 minutes. The reaction was then stopped by adding Stop Reagent and the absorbance was measured at 410 nm using a plate reader. All reagents and components were prepared according to the manufacturer's protocol and a calibration standard curve was generated using the endotoxin standard stock solutions provided in the kit (**Figure S20**).

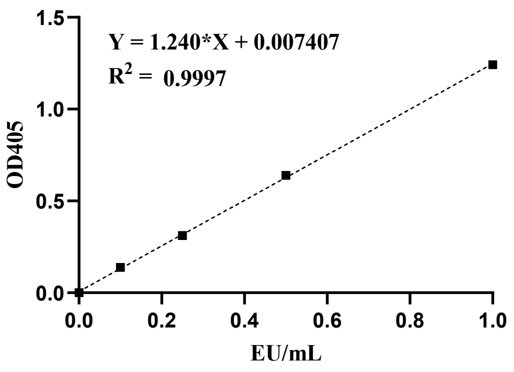

Figure S20. Calibration standard curve of endotoxin quantification.

16.6.3. Apoptosis assay

This experiment was performed using the Tali™ Apoptosis Kit - Annexin V Alexa Fluor™ 488 & Propidium Iodide and following the manufacturer's instructions. Briefly, after treatment cells were washed with PBS, trypsinised, transferred to cytometry tubes, centrifuged at 2000 rpm for 4 minutes and the supernatant removed. The pellet was resuspended in 100 µl Annexin 1X tamponade. Then 1 µl of Annexin V Alexa Fluor 488 was added to each tube and well mixed. The tubes were incubated for 20 minutes at 25°C in the dark. The cells were centrifuged, the supernatant removed and the pellet resuspended in 100 µl Annexin 1X tampon. 1 µl Tali PI was added to each sample and thoroughly mixed. Samples were incubated at 25°C for 5 minutes in the dark and analysed by flow cytometry.

16.6.4. ROS assay

This experiment was performed using the CellROX™ Green Flow Cytometry Assay Kit according to the manufacturer's instructions**.** The cellular uptake for flow cytometry was adapted following the kit instructions. After treatment, cells were washed with PBS, trypsinized, transferred to cytometry tubes, centrifuged at 2000 rpm for 4 minutes, and the supernatant was discarded. The pellet was resuspended in CellROX® Green reagent at a final concentration of 50 nM in PBS and incubated for 30 minutes in the dark. SYTOX® Red Dead Cell Stain was then added at a final concentration of 1 µM in PBS and incubated for an additional 15 minutes in the dark. Samples were immediately analyzed by flow cytometry.

16.6.5. Haemolysis Assay

In order to conduct the haemolysis assay, the procedure was carried out according to the NCL Method ITA-1 protocol for the analysis of hemolytic properties of nanoparticles.[10] As required by the protocol, the plasma-free hemoglobin (PFH) concentration had to be below 1.0 mg/mL. Whole blood was collected in heparinized tubes and diluted with PBS to adjust the total blood hemoglobin (TBH) concentration to 10 ± 2 mg/mL.A hemoglobin standard was used to build a calibration curve covering the concentration range from 0.025 to 0.80 mg/mL. The calibration standard curve was generated using serial dilutions of the hemoglobin stock solution (**Figure S21**). Separate tubes were prepared containing calibration standards, suspensions of Cu@BTTAA-Cy5-NPs (**11**), BTTAA-Cy5-NPs (**10**), and Cu@BTTAA-NPs (**7**), as well as PBS (negative control) and Triton X-100 (positive control), were each added to 700 µL of PBS. Subsequently, 100 µL of the TBH dilution (TBHd) was added to each tube and incubated at 37°C for 3 hours ± 15 minutes. To evaluate potential assay interference by the NPs, they were also incubated with PBS in the absence of blood. After incubation, the samples were centrifuged at 800 × g for 15 minutes. Then, 100 µL of the supernatant was transferred to a 96-well plate along with 100 µL of CMH reagent. The CMH reagent was prepared according to the manufacturer's instructions. Optical density (OD) was measured at 540 nm. The percentage of haemolysis was calculated using the following equation:

$$Haemolysis\ (\%) = \frac{(sample\ -\ negative\ control)}{(TBHd\ -\ negative\ control)} x100$$

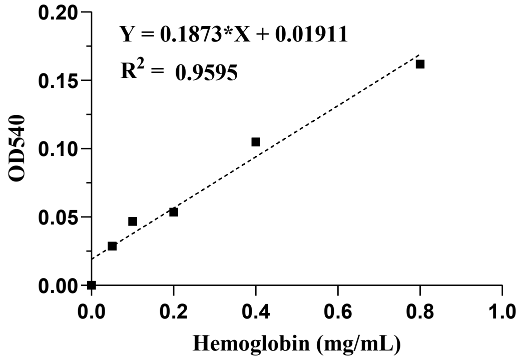

Figure S21. Calibration curve for the determination of plasma-free hemoglobin (PFH).

17. Statistical Analysis

All experiments were performed in triplicate unless otherwise stated. Data were pre-processed by removing clear outliers based on Grubbs' test (α=0.05) and checked for normal distribution using the Shapiro–Wilk test. For normally distributed data, statistical comparisons between two groups were performed using an unpaired t-test. For comparisons among multiple groups, one-way and two-way analysis of variance (ANOVA) followed by Tukey's post-hoc test was used. The significance level (α) was set at 0.05. Results are presented as mean ± standard deviation (SD), and the exact sample size (n) is indicated in the figure legends. Statistical analyses on data obtained were performed and represented with the GraphPad Prism software (version 8.0.1) (GraphPad Software, La Jolla, CA, USA).

18. References

(1) Unciti-Broceta, A.; Johansson, E. M. V; Yusop, R. M.; Sánchez-Martín, R. M.; Bradley, M. Synthesis of Polystyrene Microspheres and Functionalization with Pd0 Nanoparticles to Perform Bioorthogonal Organometallic Chemistry in Living Cells. *Nat Protoc* **2012**, *7* (6), 1207–1218. https://doi.org/10.1038/nprot.2012.052.

(2) Besanceney-Webler, C.; Jiang, H.; Zheng, T.; Feng, L.; Soriano del Amo, D.; Wang, W.; Klivansky, L. M.; Marlow, F. L.; Liu, Y.; Wu, P. Increasing the Efficacy of Bioorthogonal Click Reactions for Bioconjugation: A Comparative Study. *Angewandte Chemie International Edition* **2011**, *50* (35), 8051–8056. https://doi.org/https://doi.org/10.1002/anie.201101817.

(3) Sun, L.; Gai, Y.; Anderson, C. J.; Zeng, D. Highly-Efficient and Versatile Fluorous-Tagged Cu(i)-Catalyzed Azide–Alkyne Cycloaddition Ligand for Preparing Bioconjugates. *Chemical Communications* **2015**, *51* (96), 17072–17075. https://doi.org/10.1039/C5CC06858D.

(4) Fuentes-Cervantes, A.; Ruiz Allica, J.; Calderón Celis, F.; Costa-Fernández, J. M.; Ruiz Encinar, J. The Potential of ICP-MS as a Complementary Tool in Nanoparticle–Protein Corona Analysis. *Nanomaterials* **2023**, *13* (6). https://doi.org/10.3390/nano13061132.

(5) Zhu, J.; You, Y.; Zhang, W.; Pu, F.; Ren, J.; Qu, X. Boosting Endogenous Copper(I) for Biologically Safe and Efficient Bioorthogonal Catalysis via Self-Adaptive Metal–Organic Frameworks. *J Am Chem Soc* **2023**, *145* (3), 1955–1963. https://doi.org/10.1021/jacs.2c12374.

(6) Brenner, A. J.; Harris, E. D. A Quantitative Test for Copper Using Bicinchoninic Acid. *Anal Biochem* **1995**, *226* (1), 80–84. https://doi.org/10.1006/ABIO.1995.1194.

(7) Brenner, A. J.; Harris, E. D. Volume 226, Number 1 (1995), in the Article "A Quantitative Test for Copper Using Bicinchoninic Acid," by Andrew J. Brenner and Edward D. Harris, Pages 80–84. *Anal Biochem* **1995**, *230* (2), 360. https://doi.org/10.1006/ABIO.1995.1493.

(8) Ozkal, E.; Özçubukçu, S.; Jimeno, C.; Pericàs, M. A. Covalently Immobilized Tris(Triazolyl)Methanol–Cu(I) Complexes: Highly Active and Recyclable Catalysts for CuAAC Reactions. *Catal Sci Technol* **2011**, *2* (1), 195–200. https://doi.org/10.1039/C1CY00297J.

(9) Pourjavadi, A.; Tajbakhsh, M.; Farhang, M.; Hosseini, S. H. Copper-Loaded Polymeric Magnetic Nanocatalysts as Retrievable and Robust Heterogeneous Catalysts for Click Reactions. *New Journal of Chemistry* **2015**, *39* (6), 4591–4600. https://doi.org/10.1039/C4NJ02134G.

(10) Neun, B. W.; Cedrone, E.; Dobrovolskaia, M. A. Analysis of Hemolytic Properties of Nanoparticles. *National Cancer Institute's Nanotechnology Characterization Laboratory Assay Cascade Protocols* **2020**. https://doi.org/10.17917/V9AP-D094.
